# Self-replenishing Ni-rich stainless-steel electrode toward oxygen evolution reaction at ampere-level

**DOI:** 10.1038/s42004-025-01549-4

**Published:** 2025-05-14

**Authors:** Xiang Lyu, David A. Cullen, Max Pupucevski, Runming Tao, Harry M. Meyer, Jun Yang, Jianlin Li, Todd J. Toops, Tamara J. Keever, Hnin Khaing, Emily Tong, Judith Lattimer, Tomas Grejtak, J. David Arregui-Mena, Alexey Serov

**Affiliations:** 1 Electrification and Energy Infrastructures Division, Oak Ridge National Laboratory, Oak Ridge, TN 37831 USA; 2 Center for Nanophase Materials Sciences, Oak Ridge National Laboratory, Oak Ridge, TN 37831 USA; 3 Giner Labs, Newton, MA USA; 4 Chemical Sciences Division, Oak Ridge National Laboratory, Oak Ridge, TN 37831 USA; 5 Buildings and Transportation Science Division, Oak Ridge National Laboratory, Oak Ridge, TN 37831 USA; 6 Materials Science and Technology Division, Oak Ridge National Laboratory, Oak Ridge, TN 37831 USA; 7 Present Address: Applied Materials Division, Argonne National Laboratory, Lemont, IL 60439 USA

**Keywords:** Electrocatalysis, Hydrogen fuel

## Abstract

In the past few decades, tremendous attention has been devoted to enhancing the activity of oxygen evolution reaction (OER) catalysts for hydrogen production, while the cost and long-term stability of catalysts, which can play an even more important role in industrialization, have been much less emphasized. Herein, we engineered an OER electrode from abundant stainless steel (SS) via facile approaches, and the obtained electrode consists of a Ni-rich oxide surface layer with a Fe-rich metal substrate. An outstanding activity was observed with an overpotential of 316 mV at 100 mA cm^−2^ in 1 M KOH electrolyte. Additionally, an electrode self-replenishing concept is proposed in which a Ni-rich catalyst layer can be regenerated from a metallic substrate due to the difference in diffusion and dissolution rates of metal oxides/hydroxides, and this regeneration is validated by various characterizations. A recorded degradation rate of 0.012 was observed at 1000 mA cm^−2^ for 1000 h. The facile engineering of OER electrodes from SS combined with the self-replenishing catalyst can potentially address the cost, activity, and long-term stability barriers.

## Abstract (web summary)

While much research has been devoted to enhancing the activity of oxygen evolution reaction (OER) catalysts for hydrogen production systems, less attention has been paid to optimizing their cost and long-term stability. Here, the authors engineer an OER electrode from inexpensive and abundant stainless steel and demonstrate an electrode self-replenishing concept in which a Ni-rich catalyst layer is regenerated from a metallic substrate under OER conditions.

## Introduction

Increasing the percentage of consumed electricity, generated from renewable sources (e.g., solar, wind, tide, etc.) is critical to providing sustainable energy, mitigating a potential energy crisis, and reducing environmental impacts^1–3^. With decreasing a cost of renewable electricity, a production of basic fuels/chemical feedstocks via electrochemistry will become not only environmentally friendly but also economically favorable compared with the traditional approaches utilizing fossil fuels. The types of value-added fuels/chemicals that can be produced via electrochemistry include H_2_ from the hydrogen evolution reaction (HER) in water electrolysis^4,5^; ethylene, ethanol, methane, methanol, etc. from the CO_2_/CO reduction reaction (CO_2_/CORR)^6,7^; and ammonia from the nitrogen reduction reaction (NRR)^8,9^. Since these are all cathodic reduction reactions, the anodic oxidation reaction is essential for electrochemical balance. This is typically the oxygen evolution reaction (OER), as OER only needs water as the source and could be easily applied for large-scale electrolysis. Consequently, OER is a critical feature for various electrochemistry technologies. The development of OER electrocatalysts with low-cost manufacturing, high activity, and long-term stability is highly appealing but currently faces many challenges^10–13^.

In the past few decades, extensive efforts have been dedicated to development of active OER catalysts without expensive and rare platinum group metals (PGM) for operation in alkaline conditions. Compared to the state-of-the-art IrO_2_ and RuO_2_ OER catalysts, transition metal oxides/hydroxides exhibit promising activity^14–16^. Many studies have shown that NiFe-derived catalysts have higher activity than either Ni or Fe monometallic catalysts^17,18^, and research is ongoing to better understand this phenomenon and design an even more active/stable catalyst^19–21^. Even though the mechanism of NiFe bimetallic electrocatalyst for the improved OER activity is still being explored, it has been proven that NiFeO_x_ material with >50% Ni is one of the most active electrocatalysts for OER^22^.

Activity, stability, and cost are the three critical factors in the development of OER electrocatalysts for practical applications. To date, NiFeO_x_ nanostructured electrocatalysts were primarily synthesized through complex techniques with high energy consumption and waste generation (e.g., gas, liquid, and solid), making obtained catalysts uneconomical and with low possibility for scale-up^23–25^. Traditionally, an electrode is composed of a thin catalyst layer and ionomer loaded on a substrate (e.g., Ti, and Ni). In practice, the long-term stability of OER electrodes is always a concern, and degradation of substrate, ionomer, and electrocatalysts as whole can contribute to performance loss^26^. Substrate degradation mainly occurs via passivation, while the ionomer can degrade via both morphological and chemical routes. The electrocatalyst activity can be affected by dissolution, detachment from the substrate, and structural changes (e.g., morphology, crystal, and electronic structure) of the catalyst. Consequently, the optimization of the integration of catalysts, ionomers, and substrates is critical to developing OER electrodes with high activity and long-term stability. To date, most OER studies have been mainly focused on decreasing of the catalyst cost and enhancing the OER intrinsic activity; however, it is at least equally important to develop scalable catalyst manufacturing techniques that result in improved long-term stability at high current density^27^. Additionally, the traditional electrode design with an ultra-thin catalyst layer possesses inherent defects and cannot guarantee long-term stability due to the continuous catalyst degradation under harsh environments (e.g., high pH, electrolyte flow, O_2_ bubble formation, and a high potential at high current density)^28,29^.

Stainless steel (SS), a cheap and widely used alloy with the predominant constituents of Fe, Cr, and Ni, recently has attracted attention as a potential OER electrode, and several researchers have observed promising OER activity in alkaline media due to the NiFeO_x_ layer formation on the SS surface through facile electrochemical activations^30–36^. The electrochemical activation could induce a Ni-rich layer on the surface via the metal dissolution and redeposition. The pretreated SS materials can be used as electrodes directly with a thin NiFeO_x_ layer as an active ionomer-free catalyst matrix with a highly conductive metal backbone, respectively. Inspired by these observations and findings, we propose a self-replenishing catalyst concept on the SS electrode for OER in a way that the catalyst layer can be constantly replenished from the metal substrate under OER conditions. This feature addresses both catalyst stability and the degradation of the substrate. In this configuration ionomer can be omitted, as the NiFeO_x_ catalyst layer naturally forms on the highly conductive, ionomer-free SS metal substrate. Due to formation of nano-meter sized self-replenished catalyst layer, a consumption of bulk SS substrate is minimal (substrate features are on the level of tens of micrometers). Herein, we report the optimization of activation conditions for SS electrode preparation, and as obtained Ni-rich SS electrodes in fact demonstrate one of the best performance/durability combination among all reported NiFe-based electrocatalysts for OER. A degradation rate of 0.012 mV h^–1^ is observed at 1000 mA cm^–2^ for 1000 h, which is on a par with or better than the best reported materials. Combining the pre- and post-characterization of the SS electrodes and electrolytes, we confirm that a nm-sized NiFeO_x_ catalyst layer can be replenished from the metal substrate under harsh OER conditions. This work can potentially address all three bottlenecks of activity, stability, and cost associated with commercialization of alkaline and AEM electrolyzers.

## Results

### Optimization of activation conditions for activating SS electrodes toward OER

**Fig. 1:**
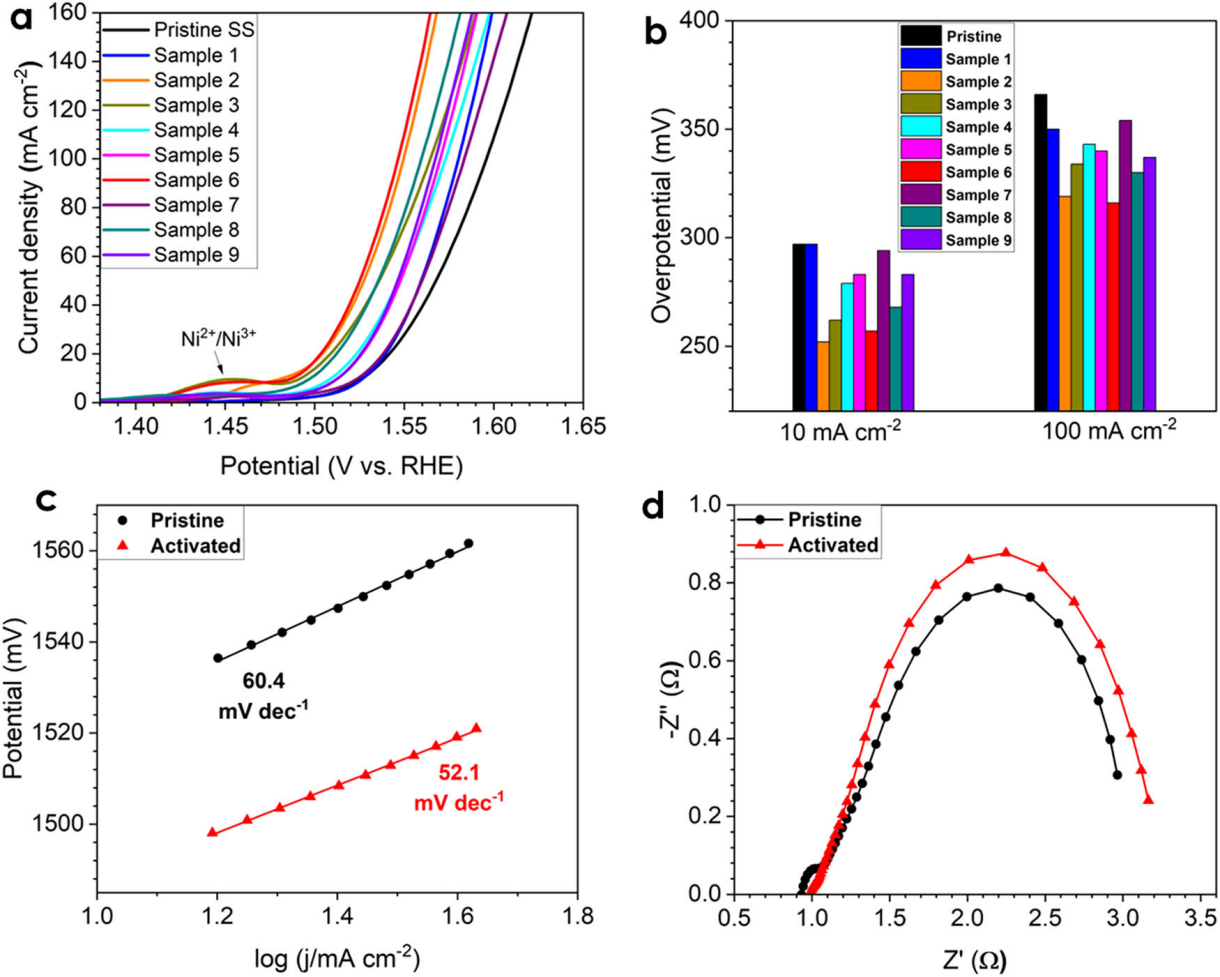
Performance evaluation of SS electrodes for OER in 1 M KOH electrolyte. **a** LSV curves of SS electrodes. **b** Corresponding overpotential at 10 and 100 mA cm^–2^, respectively. **c** Tafel slopes of the pristine and activated SS electrodes. **d** Nyquist plots of the pristine and activated SS electrodes.

To obtain an SS electrode with high activity toward OER, the activation conditions need to be optimized. Previously the effects of operating temperatures, applied current densities, operating times, and KOH concentrations on the activation of the SS electrodes were reported^30–32^. In the current study, we expanded the previously reported to include a Taguchi-based orthogonal array experimental design to reduce the number of experiments while still obtaining an optimized procedure. Each of the factors mentioned above was tested at three levels which were named, low, middle, and high, as shown in Supplementary Table 1. A total of 9 SS electrodes were pretreated under various conditions, and the weight of pristine SS shows great repeatability as shown in Supplementary Table 2. Supplementary Fig. 1 displays the chronopotentiometry (CP) curves during activation for 9 SS electrodes. Figure 1a, b displays the performance evaluation of pristine and activated SS electrodes towards OER. It can be seen that all activated SS electrodes show improved performance compared with the pristine one (Supplementary Table 3), while sample 6 exhibits the highest activity with the overpotential just 316 mV recorded at the current density of 100 mA cm^–2^, which is one of the best among all reported electrocatalysts as shown in Supplementary Table 4. According to Fig. 1a, a well pronounced peak at around 1.45 V vs. RHE was observed in activated SS electrodes, which is associated with the oxidation of Ni^2+^ to Ni^3+^ ^18,37^, while no such peak was presented in pristine electrode, indicating the surface of electrode was enriched with Ni-based species after the activation.

To avoid the influence of oxidation peak Ni^2+^/Ni^3+^ on the overpotential reported at 10 mA cm^–2^ (which is common practice in the OER research field^38^), an overpotential at 100 mA cm^–2^ was chosen for the statistical analysis and shown in Supplementary Fig. 2 and Supplementary Table 5. It was observed that the current density has a significant impact on the activity, while KOH concentration and activation time show intermediate influence, and finally, temperature exhibits the weakest effect. Based on this data, we proposed an optimal activation conditions as: middle temperature (50 °C) and KOH concentration (4 M); short activation time (2 h); and high current density (1000 mA cm^−2^), which corresponds to trial 6 (samples 6). Therefore, sample 6 was chosen for the next step of evaluation and named the activated SS electrode. To further investigate the intrinsic activity of the activated SS electrodes, the analysis of Tafel slopes and Nyquist plots of pristine and activated SS (sample 6) electrodes was performed (Fig. 1c, d, respectively). The smaller Tafel slope of the activated SS electrode indicated that the OER kinetics was enhanced, while the Nyquist plot revealed that the activated SS electrode possessed a slightly higher charge transfer resistance, which may arise from a thicker metal oxide/hydroxide layer on the electrode surface. Supplementary Fig. 3a shows the LSV curves of pristine and activated SS electrodes at high current density, and the outstanding OER improvement can be noticed from the activated SS electrode compared with the pristine one.

It is well known that the electrochemically active surface (ECSA) has a remarkable impact on the catalytic activity^39^, and the ECSA of the pristine and activated SS electrodes was evaluated as shown in Supplementary Fig. 3. We noticed that the ECSA of the activated SS electrode increased by 17.5% from 34.3 to 40.3 cm^–2^ based on Supplementary Fig. 3b, suggesting more electrochemically active sites were formed and exposed after the activation. Supplementary Fig. 3c exhibits the intrinsic activity of pristine and activated SS electrodes after normalizing by ECSA, and the activated electrode still demonstrated a higher intrinsic activity compared with the pristine one. Therefore, the high activity of the activated SS electrode is attributed to both the highly active catalyst species generation and the increase of ECSA after the activation.

**Fig. 2:**
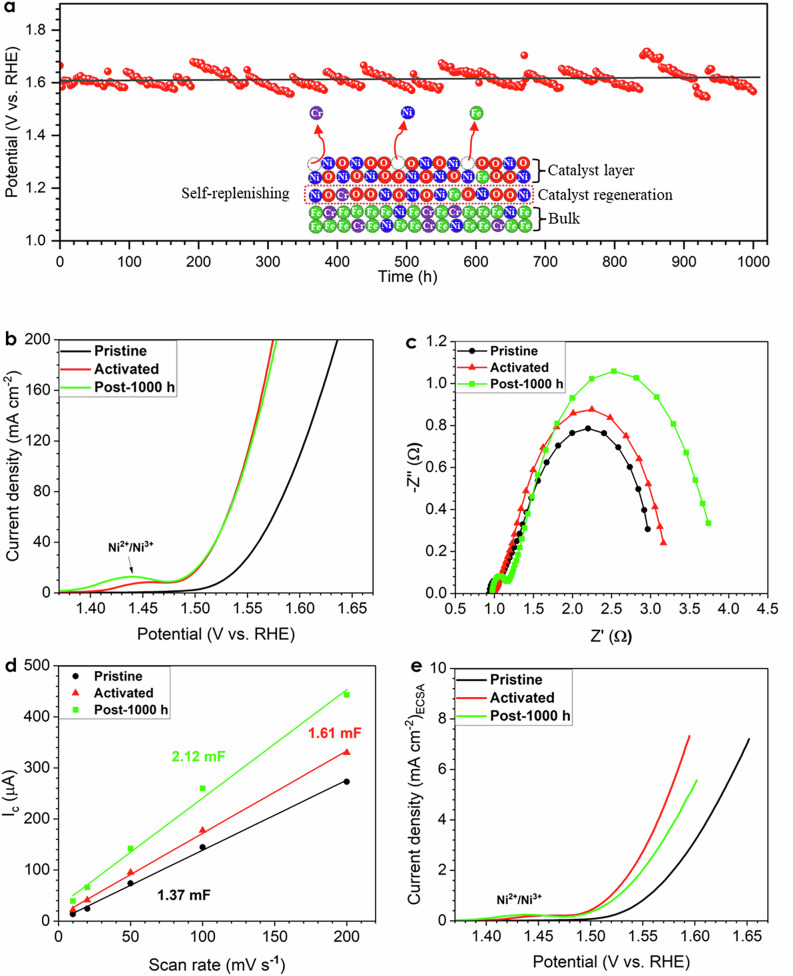
Durability test and activity evaluation. **a** Durability test in a three-electrode system at 1000 mA cm^–2^, around 22 °C in 1 M KOH electrolyte. **b** LSV curves and (**c**) Nyquist plots of pristine, activated, and post-1000 h SS electrodes. **d** ECSA measurement and (**e**) normalized LSV curves based on the ECSA of pristine, activated, and post-1000 h SS electrodes.

Figure 2a displays the long-term durability test with the activated SS electrode at 1000 mA cm^–2^ in the three-electrode system, with a temperature around 22 °C in 1 M KOH electrolyte. We can observe a regular small fluctuation of the potential as a function of time, as the electrolyte was consumed during the electrolysis process and fresh DI water needed to be added in order to maintain the KOH concentration, which resulted in an insignificant fluctuation of the KOH concentration, later led to the potential fluctuation. The total overpotential increased by 12 mV in 1000 h with a degradation rate of 0.012 mV h^–1^, making this material on of the most stable ones, based on the exhaustive literature review. A LSV was performed on the electrode after 1000 h using fresh 1 M KOH as shown in Fig. 2b, and the activity was comparable with the electrode before the durability test. However, an obvious increase in the charge transfer resistance was noted after the 1000 h durability test as shown in Fig. 2c, which may be the indication of the SS surface reconstruction and formation of a thicker metal oxide/hydroxide layer. To further investigate the intrinsic activity of the SS electrode after the durability test, the ECSA and normalized activity of the post-1000 h electrode are presented in Fig. 2d, e, respectively, and it can be seen that the ECSA further increased, while the intrinsic activity of the electrode slightly decreased after the durability test. In summary, the influences of charge transfer resistance and ECSA may offset each other during durability experiments for maintaining a high activity. To elucidate the activity enhancement after activation and roots of outstanding durability, various characterizations of pristine, activated, post-1000 h durability SS electrodes, metal dissolution into electrolyte, as well as metal deposition onto the surface of counter electrode (CE) were performed.

### Characterization of pristine, activated, and post-durability SS electrodes

Supplementary Fig. 4 shows the XRD patterns of pristine, activated, post-1000 h durability SS electrodes. Peaks of 111, 200, and 220 crystal phases were observed from all evaluated SS electrodes^40,41^, while no obvious difference was noticed based on XRD patterns. Supplementary Fig. 5 displays the SEM image of three SS electrodes revealing no obvious difference among those three electrodes. The surface roughness increased after the activation and durability, which increased the ECSA of activated and post-1000 h durability SS electrodes. To understand the element distribution change on SS electrode surface, the SEM/EDS measurement was performed, and Supplementary Fig. 6 shows an SEM/EDS representative example of the pristine SS electrode surface. Supplementary Fig. 7 summarizes all detected elements for three electrodes except carbon, as carbon contamination can be introduced from the environment. It can be seen that the predominant metals were Fe, Cr, and Ni, which is consistent with the 316 SS composition^42^. In addition, no apparent difference in metal composition change was observed among those three electrodes if we consider the statistical error bars. Interestingly, an O content increased after the durability test, indicating that SS surface was oxidized during the durability test. Since we mainly focus on the Fe, Ni, and Cr elemental distributions on the SS surface, those three element distributions were summarized in Supplementary Fig. 8. No obvious difference among Fe, Cr, and Ni distributions was noticed when considering error bars, indicating that both activation and durability processes did not change the bulk metal composition.

**Fig. 3:**
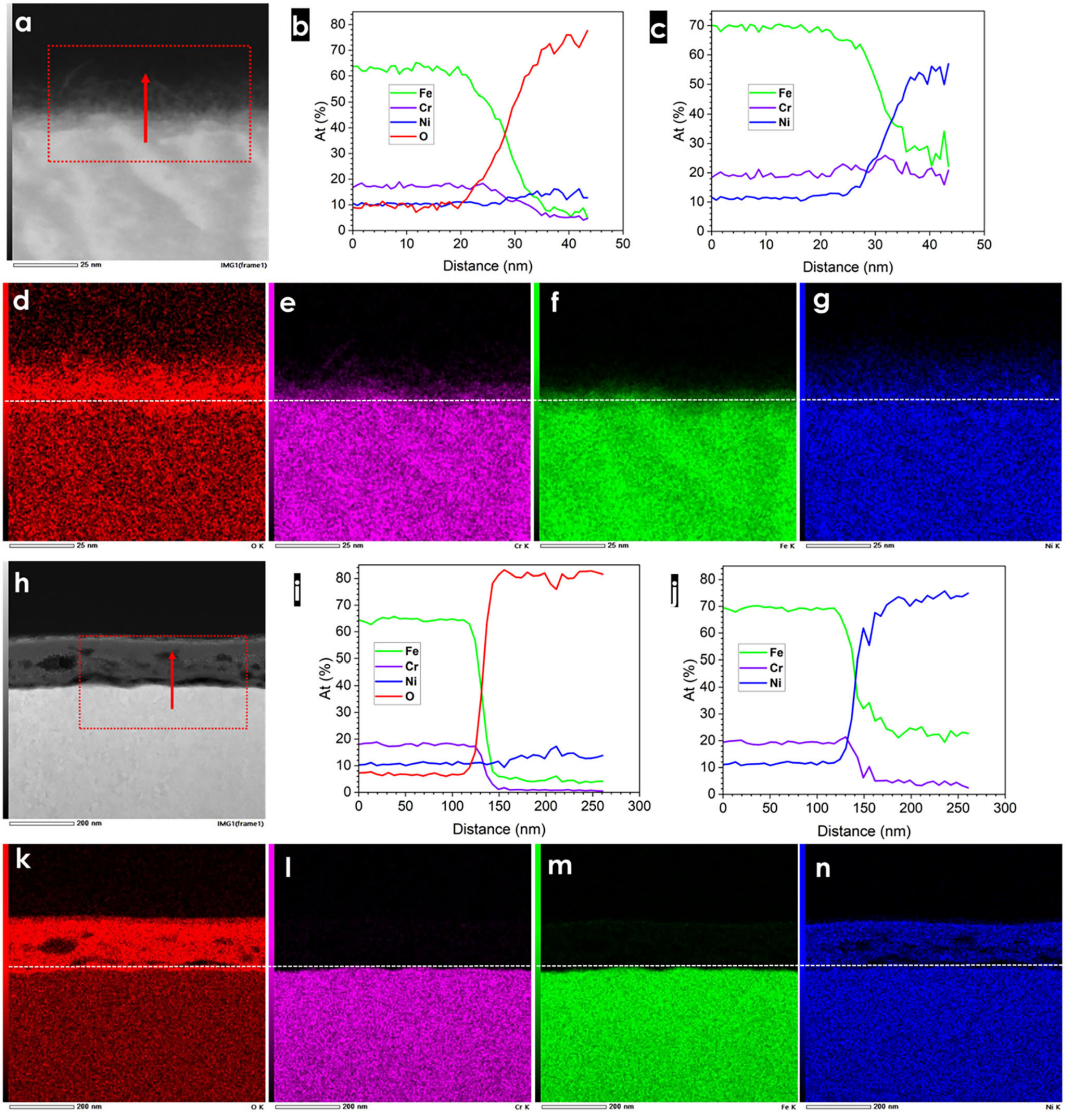
STEM/EDS line scanning and images of activated and post-1000 h SS electrodes. **a** SEI image of activated SS electrode for EDS scanning with direction. **b**, **c** Line scanning element distribution with and without O. **d–g** O, Cr, Fe, and Ni STEM/EDS images, respectively. **h** SEI image of post-1000 h SS electrode for EDS scanning with direction. **i**, **j** Line scanning element distribution with and without O. **k–n** O, Cr, Fe, and Ni STEM/EDS images, respectively.

To investigate the SS surface change further, STEM-EDS cross-section characterization with focused ion beam (FIB) was carried out on the activated and post-1000 h durability SS electrodes (Fig. 3 and Supplementary Fig. 9). According to the high-angle annular dark-field (HAADF)-STEM cross-section images Supplementary Fig. 9a–c, a clear catalyst layer with a thickness of around 20 nm was observed, and this catalyst layer exhibited a multi-crystalline structure. In contrast, a porous catalyst layer with around 150 nm was noticed on the post-1000 h SS electrode surface as shown in Supplementary Fig. 9d–f, indicating a much thicker catalyst layer was formed after the durability test, which is consistent with charge transfer resistance analysis.

Figure 3a, d, g shows the STEM/EDS mappings of activated SS electrode at 20 nm scale, and the O signal was very strong in the catalyst layer followed by Ni, indicating that O and Ni were enriched in those areas. To understand better the element distribution in the catalyst layer, a STEM/EDS line profile was performed as shown in Fig. 3b, c. In the metal substrate, the Fe content is the highest with ~62 at.% having Ni lowest content of about 10 at.%. The Fe and Cr contents had an opposite trend to those of Ni and O in area approaching the catalyst layer, which is more pronounced in case of O (Fig. 3b). These results suggest that a Ni/Fe (oxy)hydroxide/oxide catalyst layer was formed on the activated SS electrodes with enrichment of Ni. In addition, the results show that Fe and Cr dissolution rates are much faster compared to Ni under activation conditions, forming a Ni-rich catalyst layer. According to the Pourbaix (E-pH) diagrams of Fe-Cr-Ni alloy^43^, Ni tends to form stable oxide/hydroxide complexes at high potential and pH, while Fe and Cr exhibit an opposite trend forming soluble compounds. Consequently, Fe and Cr compounds prone to faster dissolution than Ni during the activation, leading to formation a Ni-rich catalyst layer. Figure 3c displays the STEM/EDS line profile plotting only Fe, Cr, and Ni metals, and it can be seen that Ni content increased while Fe dropped, having Cr concentration relatively stable.

Figure 3h, k, n presents the STEM/EDS mappings of post-1000 h SS electrode at 200 nm scale, and O and Ni were enriched of the catalyst layer, which is similar to the activated SS electrode. Figure 3i, j displays the corresponding STEM/EDS line profile and the similar activated SS electrodes trend was observed, showing an increase of O and Ni as well as decrease of Fe and Cr concentration. A slight difference was noticed in Fig. 3j, that Cr content dropped instead of being stabilized as in a case of activated SS electrode, indicating more Cr was dissolved in the durability test than during the activation process.

**Fig. 4:**
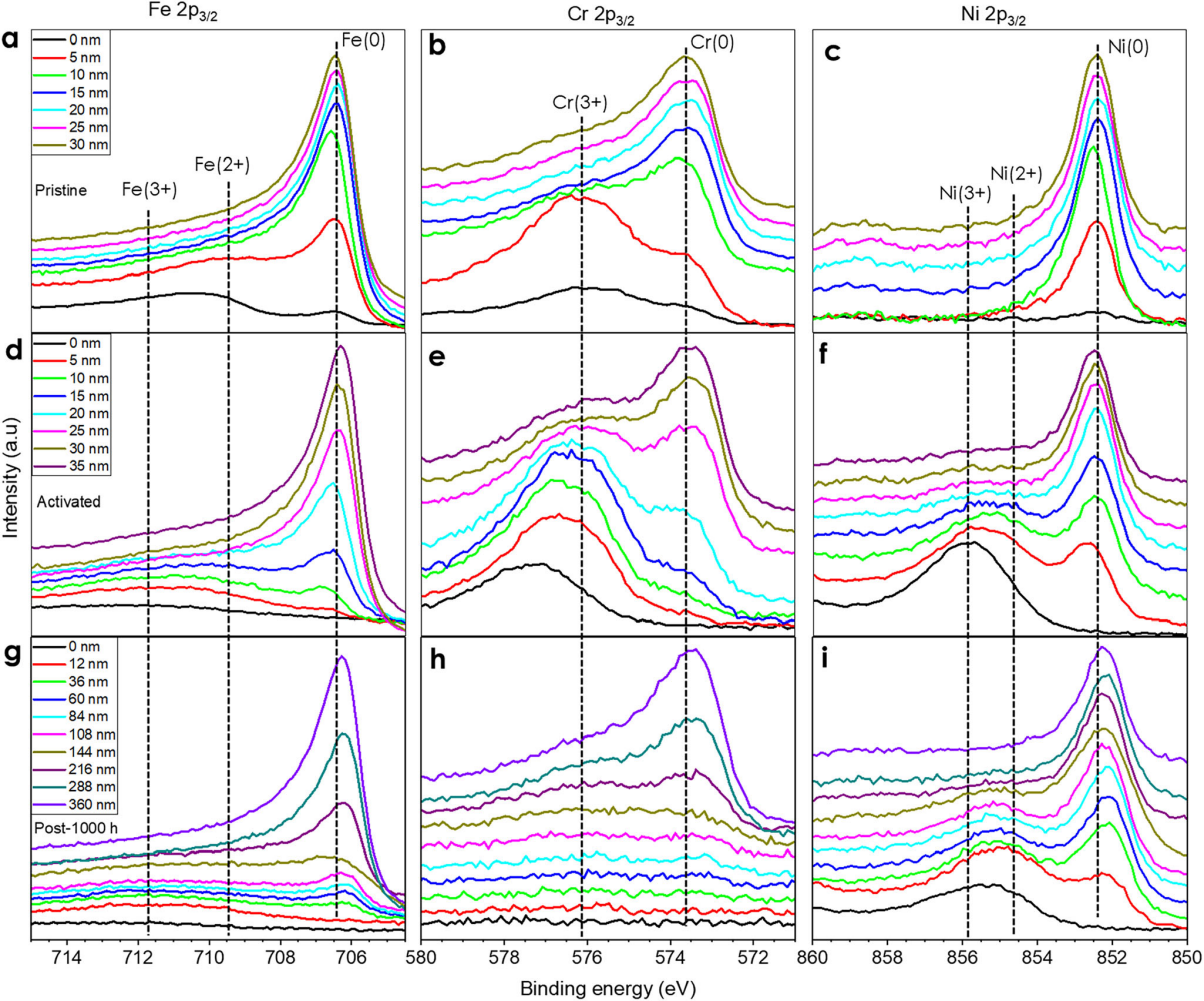
High resolution of XPS depth profile characterization of SS electrodes. **a** Fe 2p_3/2_, (**b**) Cr 2p_3/2_, and (**c**) Ni 2p_3/2_ for the pristine SS electrode. **d** Fe 2p_3/2_, (**e**) Cr 2p_3/2_, and (**f**) Ni 2p_3/2_ for the activated SS electrode. **g** Fe 2p_3/2_, (**h**) Cr 2p_3/2_, and (**i**) Ni 2p_3/2_ for the post-1000 h SS electrode.

To elucidate the metal oxidation state on SS electrode surface, an XPS depth profile characterization was performed on the pristine, activated, and post-1000 h electrodes (Fig. 4 and Supplementary Figs. 10, 11). According to the XPS depth survey (Supplementary Fig. 11), the O content was high in all three electrodes. The O content was higher than 30 at% in the 10 nm depth for the pristine SS electrode, while it went to the depth of around 35 nm for the activated SS electrode, indicating metal oxide/hydroxide was formed on the SS electrode surface during the activation. In comparison, the O content was higher than 30 at% until around 350 nm depth for the post-1000 h SS electrode, suggesting that much more metal oxide/hydroxide was generated on the SS surface in the durability test. The results indicate that the thickness of formed metal oxide/hydroxide layer grows significantly after the durability test. In addition, similar to the STEM/EDS line scanning, O and Ni content increased with the decrease of Fe and Cr from the bulk to the surface of the activated and post-1000 h SS electrodes. According to Supplementary Fig. 11a–c, the Ni content increased from 6 to 62 at% in the pristine and activated SS electrodes, respectively, while the Fe content decreased from 70 to 26 At% correspondingly, further confirming the formation of the Ni-rich catalyst layer after the activation process. Additionally, the surface metal distribution on the post-1000 h electrode (Supplementary Fig. 11f) only insignificantly changed in comparison with the activated electrode, with the atomic ratio of Ni/Fe around 7/3 in both activated and post-1000 h electrodes. Interestingly, numerous researchers have reported that electrocatalysts with a Ni/Fe atom ratio of around 7/3 show the highest activity toward OER^44–47^, which is coincidently in line with our results.

Figure 4 displays the high-resolution XPS depth profile characterization of Fe 2p_3/2_, Cr 2p_3/2_, and Ni 2p_3/2_. In general, we can observe that all peaks shifted positively to higher energy after the activation process, indicating that the valence state of all three metals increases. Even stronger peak at high binding energy was observed at high depth after the durability test, suggesting that more metal oxide/hydroxide on the activated SS surface, which is consistent with the element distribution from the STEM/EDS characterization aforementioned. Detailed analysis of the spectra of the pristine SS electrode indicates that, the Fe 2p_3/2_ peak (Fig. 4a) on the surface corresponded to Fe oxide peak as well as a very weak Fe metallic one. It shifted to a lower energy with the predominant Fe metallic peak at the depth of 5 nm, and only a strong Fe metallic peak was observed at 10 nm depth. In contrast, a weak Fe oxide peak was still the major constituent at 10 nm, and a weak metallic Fe was the primary peak until 15 nm depth for the activated SS as shown in Fig. 4d. Additionally, both Fe oxide and metallic peaks were very weak until 144 nm depth for the post-1000 h SS electrode (Fig. 4g) as an Fe content decreased significantly on the surface after the durability test. Similar trends were noticed for Cr 2p_3/2_ spectra (Fig. 4b, e, h), and the only difference is that Cr only possesses tri-valence oxidation state and Cr is easier to be oxidized than Fe, leading to tri-valence oxidation state of Cr was still prime at 20 nm in case of activated SS electrode. The easy oxidation of Cr may arise from the lower electronegativity value of Cr compared to Fe.

In contrast, only Ni metallic state was observed from the pristine SS electrode (Fig. 4c), while it shifted to higher energy associated with the Ni^3+^ oxidation state on the surface for both activated and post-1000 h SS electrodes. The metallic Ni as the primary state was observed at 10 and 60 nm for the activated and post-1000 h SS electrodes, respectively. These facts indicate that among the Fe, Cr, and Ni metals, Ni is the metal least prone to oxidation, which may arise from its highest electronegativity. Both Fe^3+^ and Ni^3+^ oxidation states are observed after the activation as well as for post-1000 h SS electrodes. Based on that they can be considered as the OER active sites^48–50^. In summary of the XPS analysis, (1) a Ni-rich catalyst layer was formed on the activated SS electrode surface and it maintained on the post-1000 h SS electrode; (2) the metal oxidation ability follows this trend Cr > Fe > Ni; (3) metals were oxidized deeper and to a higher oxidation state in the activated SS electrode; (4) metals were oxidized much further in the post-1000 h SS electrode, which is highly related to the catalyst replenishment. It should be mentioned that the catalyst layer thickness from XPS measurement was around 2 times that from cross-section STEM. The depth determined by XPS is based on a sputter rate measured using standard films of SiO_2_, and the depths are only accurate if the oxides of Fe, Ni, etc. sputtered at the same rate as the SiO_2_. However, that is likely not the case most of the time, and the depth error can be significant in the case of XPS. Thus, the catalyst layer thickness measurement from cross-section STEM is more accurate.

### Investigation of metal dissolution during activation and durability

The metal dissolution from an electrode is critical to evaluate catalyst durability and can also validate the catalyst replenishment process of this study. Therefore, amount of metals including Fe, Cr, and Ni dissolved into electrolytes and deposited on the CE during activation and durability test were quantified and shown in Supplementary Tables 6–8 and Supplementary Fig. 13. We can observe that all three metals are detected from the CE surface in both activation stage and durability test (Supplementary Table 6), while only Fe and Cr were found in the electrolyte during the activation process (Supplementary Table 7). Nickel was not observed until 1000 h in the durability test. The results imply that both metal deposition on CE and metal dissolution in electrolyte are vital for catalyst durability evaluation, and measurement of metal dissolution in electrolyte alone is insufficient.

Supplementary Table 8 summarizes the total metal dissolution, and we can see that total metal loss during 2 h activation process (237.7 µg) was significantly higher than that after 1000 h durability (163.8 µg). Specifically, Fe dissolved the most, followed by Cr, and then Ni for both activation and durability processes. Additionally, the dissolution ratios of Fe/Ni and Cr/Ni are 29.5 and 8.6, respectively, while the dissolution ratio of Fe/Cr is around 3.4 during an activation process. According to the SEM/EDS characterization, the metal weight ratios of Fe/Ni, Cr/Ni, and Fe/Cr in the electrodes are 5.6, 1.4, and 3.9, respectively, indicating that the dissolution rate of Fe is slightly lower than that of Cr but both are much faster than that of Ni. This also supports the formation of the Ni-rich catalyst layer during the activation. Similar trend was observed after durability test, and the dissolution rates of Fe and Cr were higher than that of Ni, while the dissolution rate of Fe is slightly higher than that of Cr. The results confirm a Ni-rich catalyst layer was replenished in the durability test, which is in agreement with a thickness increase of a catalyst layer and a promotion of metal oxidation. A total metal loss of 163.8 µg after 1000 h durability test was only 0.06% compared with the total electrode weight (266.1 mg), indicating that the formed Ni-rich catalyst layer possesses extreme stability.

Additionally, a metal mass detected from CE and electrolyte was compared as shown in Supplementary Fig. 12, and a total mass detected from CE was comparable with that from the electrolyte after 2 h activation process. Specifically, most of Fe was detected from the CE but the Cr exhibited the opposite trend, and the Ni was only observed from the CE. In contrast, the total mass detected from the CE was more than 10 times higher compared with that from the electrolyte, and all three metals detected from the CE were higher than that from the electrolyte after the durability test. These results indicate that metal dissolution rates of metal are not constant during the stability test, and the dissolution rate is high at the beginning while decreasing with time, which can confirm the outstanding stability of the Ni-rich catalyst layer as well. Recently, Tyndall et al. identified and proved the source of such inherent instability of the NiFeO_x_ layered double hydroxide (LDH) catalyst for OER, and revealed that measurably higher leaching rates of Fe metals compared to Ni come from highly active edge sites and is thermodynamic driving force for increased leaching of Fe metals^28^, which is consistent with findings in this study. The results imply that catalyst replenishment from a metal substrate to self-repair the catalyst layer can be an effective approach to address a catalyst stability issue.

### Long-term stability in the anion exchange membrane water electrolyzer (AEMWE)

**Fig. 5:**
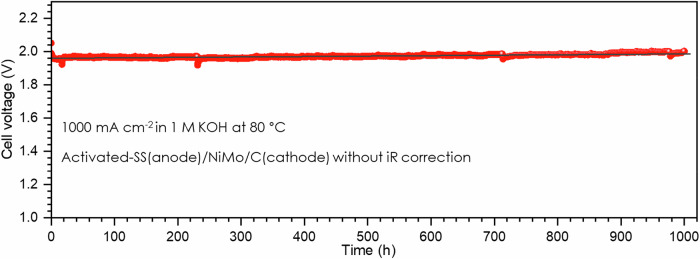
Cell voltage measured at current density of 1000 mA cm^–2^ in AEMWE. The cell was maintained at 80 °C with 1 M KOH electrolyte flowing on the anode side, and the cathode was NiMo/C catalyst loaded on a carbon paper and maintained dry.

The ultimate test of an activated SS is its performance in the operating AEMWE. The activated SS electrode was scaled up to 5 cm^2^ with the optimal activation process and was assembled into the anode of an AEMWE single cell and tested using 1 M KOH as a feed in the anode at 80 °C, and the cathode with PGM-free, NiMo/C catalyst loaded on the carbon paper was kept dry. Supplementary Fig. 13 shows the current-voltage polarization curve measured in the AEMWE, and the electrolyzer reached current densities of 1000 and 2000 mA cm^–2^ at voltages of 1.93 and 2.11 V without the iR correction, respectively, which is very promising finding for 3D electrode without any additional catalyst added. Figure 5 displays the AEMWE stability test at 1000 mA cm^–2^ for 1000 h, and a total voltage increased by 27 mV from 1960 to 1987 V in 1000 h based on statistics analysis. Thus, the degradation rate was 0.027 mV h^–1^, which was excellent, however was around 2 times compared with the three-electrode system test (0.012 mV h^–1^) at the same current density in OER test in 1 M KOH electrolyte. The reason is the AEMWE system is much more complex than the three-electrode system, and the degradation rate obtained from AEMWE includes the performance degradation of other membrane electrode assembly components, such as membrane, ionomer and cathode material, while the degradation rate obtained from the three-electrode system only refers to the anode itself. We observed that the metals could dissolve from the SS electrode to the electrolyte and then deposit on the CE in the three-electrode system, so the metal (e.g., Fe, Cr, and Ni) contamination on the membrane and cathode could lead to the performance degradation in the AEMWE.

## Conclusions

In summary, we optimized the facile electrochemical activation conditions of SS electrodes to obtain an electrode with the highest activity toward OER and found that current density and KOH concentration exhibit significant influence on the electrode activity. A NiFeO_x_ catalyst layer with Ni as the main component was formed during an activation stage and overpotentials of 257 and 316 mV were achieved from the optimum electrode at 10 and 100 mA cm^–2^ in 1 M KOH electrolyte, respectively. We proposed a self-replenishing catalyst concept on the SS electrode that Ni-rich catalyst layer can regenerate itself from a metal substrate under the OER conditions due to the different dissolution rates of Fe, Cr, and Ni, which was confirmed by the catalyst layer regeneration in the durability test. In detail, the NiFeOx catalyst layers with Ni-rich was formed due to the much higher dissolution rate of Fe and Cr compared with Ni in the activation process. In the stability test, all metals (Fe, Cr, and Ni) continued dissolved, while more Fe and Cr were dissolved than Ni. The outmost catalyst layer was depleted, and new NiFeOx catalyst layers with Ni-rich was regenerated from the Fe-rich metal substrate, which is similar to the activation process. A recorded degradation rate of 0.012 mV h^–1^ was observed at 1000 mA cm^–2^ for 1000 h, and the catalyst layer surface composition was preserved with catalyst layer thickness increase combined with a promotion of metal oxidation during durability test. A total metal dissolution was only 0.06 wt% of the electrode weight after 1000 h, implying that self-replenishing catalyst potentially can solve the long-term stability of OER catalysts. Additionally, an outstanding stability of the activated SS electrode was confirmed by AEMWE tests, and a degradation rate of 0.027 mV h^–1^ was obtained at 1000 mA cm^–2^ for 1000. Based on the activity, cost, and long-term stability required in practical applications reported here, facile engineering of self-replenishing Ni-rich SS electrodes can revolutionize a whole electrode design paradigm.

## Methods

### Activation of stainless-steel (SS) electrodes

Porous 316 SS from Mott with an area of 1 cm^2^ (1.0 × 1.0 cm) each was used. The weight of all pristine SS electrodes was measured to make sure the repeatability. For the activation, an OER occurs at the SS anode in alkaline conditions to achieve a Ni-rich surface. The electrolyte was prepared with KOH pellets (85 + %, ACS reagent, Sigma-Aldrich) and deionized (DI) water. A Pt gauze (99.9% metals basis, Alfa Aesar, 52 mesh woven from 0.1 mm diameter wire) with a geometric area of 6.25 cm^2^ was employed as the cathode for hydrogen evolution reaction (HER) during the activation. A homemade cell made of PTFE material was used, and the temperature of the electrolyte was controlled by oil bath. Additionally, the actual temperature of the electrolyte was monitored by an Infrared (IR) thermometer.

### Performance evaluation of activated SS electrodes

The performance of activated SS electrodes after activation was evaluated by the same Pine potentiostat. The same Pt mesh electrode was used as the counter electrode (CE) for HER, and a hydrogen reference electrode (Gaskatel with SKU number 81010) was employed. The electrolyte was 1 M KOH with a pH of 13.8, and an alkaline resistance cell (Pine, AF01CKT1001) was used except for the high temperature evaluation. In the performance evaluation at elevated temperatures, the same homemade PTFE cell was used. Linear sweep voltammetry (LSV) measurements were carried out at a scan rate of 10 mV s^–1^ and repeated at least 5 times to ensure repeatability. The Tafel slope plot was obtained based on the LSV data with the same current density range for comparison, as a Tafel slope may change within a current density range. The electrochemically active surface (ECSA) of SS electrodes was estimated by electrical double-layer capacitance (C_dl_), which is described in detail in our previous work^51^. The iR correction was applied to all potentials with the resistance obtained in electrochemical impedance spectroscopy (EIS), and EIS measurement was performed from 100 K Hz to 1 Hz. All potentials reported in this work are against of reversible hydrogen electrode (RHE, throughout the text) scale.

### XRD measurements

X-ray diffraction (XRD) patterns of samples were measured on a Panalytical Empyrean diffractometer at an operation voltage of 45 kV and a current of 40 mA, and obtained XRD patterns were analyzed by HighScore Plus from Malvern Panalytical.

### SEM-EDS characterization

SEM and EDS characterization were performed on a ThermoFisher Scientific Scios 2 Dual Beam Focused Ion Beam at 5 and 15 kV, respectively. For the EDS analysis, each sample was repeated at least 3 times from individual areas to obtain the average value and standard deviation.

### STEM-EDS characterization

Thin foil samples used for STEM-EDS characterization were prepared using a focused ion beam (FIB) FEI Versa 3D dual-beam FIB-SEM instrument. This method of sample preparation allows for the selection of a specific area of interest. To start the sample preparation, a section of the SS sample was covered with ink to minimize the damage produced by the ion beam. The following steps consist in reducing the size of the sample and extracting it from the bulk sample. Then the sample is welded into a grid and thinned with an ion beam into at thickness smaller than 100 nm. To further treat the thin foils, a Fischione Model 1040 NanoMill was used. This instrument works by generating a low-energy argon ion beam that removes part of the ion damage or ion implantation that may have occurred during the initial sample preparation. The samples were prepared at temperatures around –165 °C to minimize the damage to the sample. Firstly, the samples were milled at low incident angles of ±10°, using an energy of 900 eV and an emission current of 170 μA for 3 min. Subsequently, the same procedure was followed at a lower energy of 600 eV for 2 min. STEM-EDS measurements were carried out in a probe-corrected JEOL JEM-ARM200F “NEOARM” operated at 200 kV and equipped with dual 100 mm^2^ silicon drift detectors (SDD) for EDS analysis. The STEM/EDS spectrum images were quantified using the standardless routine in the JEOL Analysis Station software.

### XPS measurements

XPS was performed using a Thermo Scientific (Waltham, MA, USA) Model K-Alpha XPS instrument. The instrument utilizes monochromated, micro-focused, Al K_α_ X-rays (1486.6 eV) with a variable spot size (i.e., 30–400 µm). Analyses of the sample was performed with the 400 µm X-ray spot size for maximum signal and to obtain an average surface composition over the largest possible area. The instrument has a hemispherical electron energy analyzer equipped with a 128-channel detector system. Base pressure in the analysis chamber is typically 2 × 10^–9 ^mbar or lower. Samples were prepared for analysis by attaching the samples directly the XPS holder using metal clips. Survey spectra (pass energy = 200 eV) were acquired for qualitative and quantitative analysis and high-resolution core level spectra (pass energy = 50 eV) were acquired for detailed chemical state analysis. An Ar-ion gun operated at 2 kV and an ion current sufficient to produce a sputter rate of 12 nm min^–1^ as measured on 100 nm SiO_2_ standard films. All spectra were acquired with the charge neutralization flood gun turned on to maintain a stable analysis condition. The flood gun uses a combination of low energy electrons and argon ions for optimum charge compensation. The typical pressure in the analysis chamber with the flood gun operating is 2 × 10^–7 ^mbar. Data were collected and processed using the Thermo Scientific Avantage XPS software package (v.5.96). Peak fitting was performed using mixed Gaussian/Lorentzian peak shapes and a Shirley/Smart type background, and the calibration was done by shifting the peaks relative to the adventitious C 1 s at the binding energy of 284.8 eV.

### ICP-MS measurements

The samples were diluted using 2% nitric acid/1% hydrochloric acid matrix (Optima® Fisher Chemical™). The variable molarity KOH samples were diluted to the same matrix [K]_conc_ in the acid solution. The analysis of Fe, Cr and Ni were done via Inductively Coupled Mass Spectrometry (ICP-MS) using the quadrupole model iCAP-RQ (ThermoFisher Scientific, Bremen) in Standard mode, utilizing QTegra® ISDS software and linear regression. The responses of Fe, Cr, and Ni were provided by commercially available standards (Inorganic Ventures, Christiansburg, VA). The matrix and stability of the samples were monitored, and corrected for, using an internal standard addition, In-115. Nebulizer, uptake peristaltic pump tubing, sample cones and insert-ready skimmer cones were obtained from Elemental Scientific Inc. (Omaha, NE). Each sample was tested five times, and the average is reported here.

### Membrane electrode assembly (MEA) and anion exchange membrane water electrolyzer (AEMWE) testing

The simplified architecture of the activated SS electrode was evaluated by assessing its durability in a fully PGM-free 5 cm^2^ AEMWE at 1 A cm^–2^ for 1000 h. Prior to assembly, the membrane and electrodes were soaked in 1 M KOH for at least 2 h to exchange the membrane and cathode ionomer from the bicarbonate to the active hydroxide form. The membrane electrode assembly (MEA) consisted of an 80 μm Versogen PiperION anion exchange membrane sandwiched between the bare activated SS PTL and a Freudenberg H23C6 carbon fiber paper gas diffusion layer (GDL) coated with a PGM-free NiMo/C (50 wt% metal) catalyst with 1 mg cm^–2^_NiMo_ loading^52^. Commercially available 5 cm^2^ Scribner hardware (consisting of end plates, current collectors, and flow fields) was used as the cell housing. The cell was primed by heating to 80 °C with a recirculated 3 mL min^–1^ 1.0 M KOH feed to the anode, keeping the cathode dry. Operating in constant current mode, the cell was ramped up from 0.01 to 1 A cm^–2^ over 15 min. Each current density (0.01, 0.05, 0.1, 0.2, 0.3, 0.4, 0.5, 0.6, & 1.0 A cm^–2^) was held until the voltage appeared stable. There was an initial break-in period of ~20 h at 1 A cm^–2^, after which the cell stabilized at ~1.960 V, and a beginning of life polarization curve was taken from 0.05 to 2 A cm^–2^. The cell was then held at 1 A cm^–2^ for >1000 h. Throughout the test, periodic replenishment of the electrolyte reservoir with DI water was performed to maintain an electrolyte concentration of 1.0 M KOH.

## Supplementary information

Supplementary Material

## Data Availability

Any relevant data are available from the corresponding authors upon reasonable request.
