# Supplementary material for: Self-replenishing Ni-rich stainless-steel electrode toward oxygen evolution reaction at ampere-level
Source: Commun Chem. 2025 May 14;8:150. doi: 10.1038/s42004-025-01549-4 (PMC12078717; doi:10.1038/s42004-025-01549-4)
Supplement: Supplementary file 1 — Supplementary Material [file 42004_2025_1549_MOESM1_ESM.pdf]

**Electronic Supporting Information**

# Self-replenishing Ni-rich stainless-steel electrode toward oxygen evolution reaction at ampere-level

Xiang Lyu[1*], David A. Cullen[2], Max Pupucevski[3], Runming Tao[1†], Harry M. Meyer III[4], Jun Yang[1], Jianlin Li[1†], Todd J. Toops[5], Tamara J. Keever[4], Hnin Khaing[4], Emily Tong[3], Judith Lattimer[3], Tomas Grejtak[6], J. David Arregui-Mena[6], and Alexey Serov[1*]

[1]Electrification and Energy Infrastructures Division, Oak Ridge National Laboratory; Oak Ridge, TN 37831, USA

[2]Center for Nanophase Materials Sciences, Oak Ridge National Laboratory, Oak Ridge, TN, 37831, USA

[3]Giner Labs, Newton, MA, USA

[4]Chemical Sciences Division, Oak Ridge National Laboratory, Oak Ridge, TN 37831, USA

[5]Buildings and Transportation Science Division, Oak Ridge National Laboratory; Oak Ridge, TN 37831, USA

[6]Materials Science and Technology Division, Oak Ridge National Laboratory; Oak Ridge, TN 37831, USA

[†]Current address: Applied Materials Division, Argonne National Laboratory, Lemont, IL, 60439, USA

Corresponding authors: Xiang Lyu, email: lyux@ornl.gov Alexey Serov, email: serova@ornl.gov

Table of contents:

Supplementary Figures………………………………………………………… 2

Supplementary Tables……………………………………………………… 15

Supplementary References ……………………………………………… 20

**Supplementary Figures**

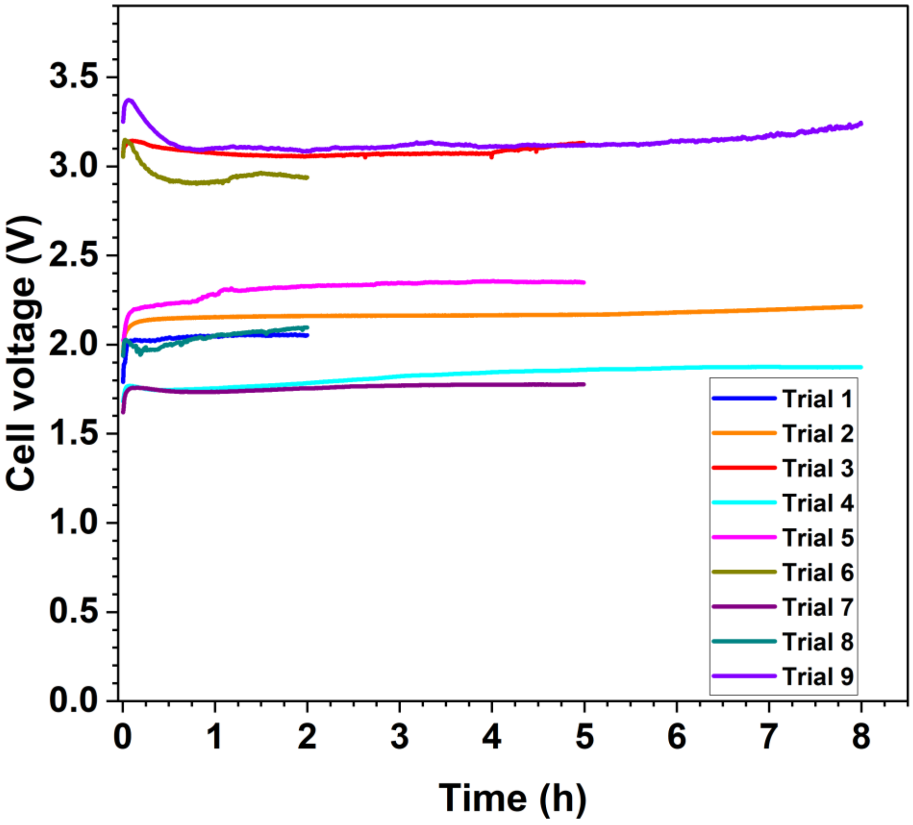


**Supplementary Figure S1**. The chronopotentiometry (CP) curves during activations for 9 SS electrode samples, without iR correction.

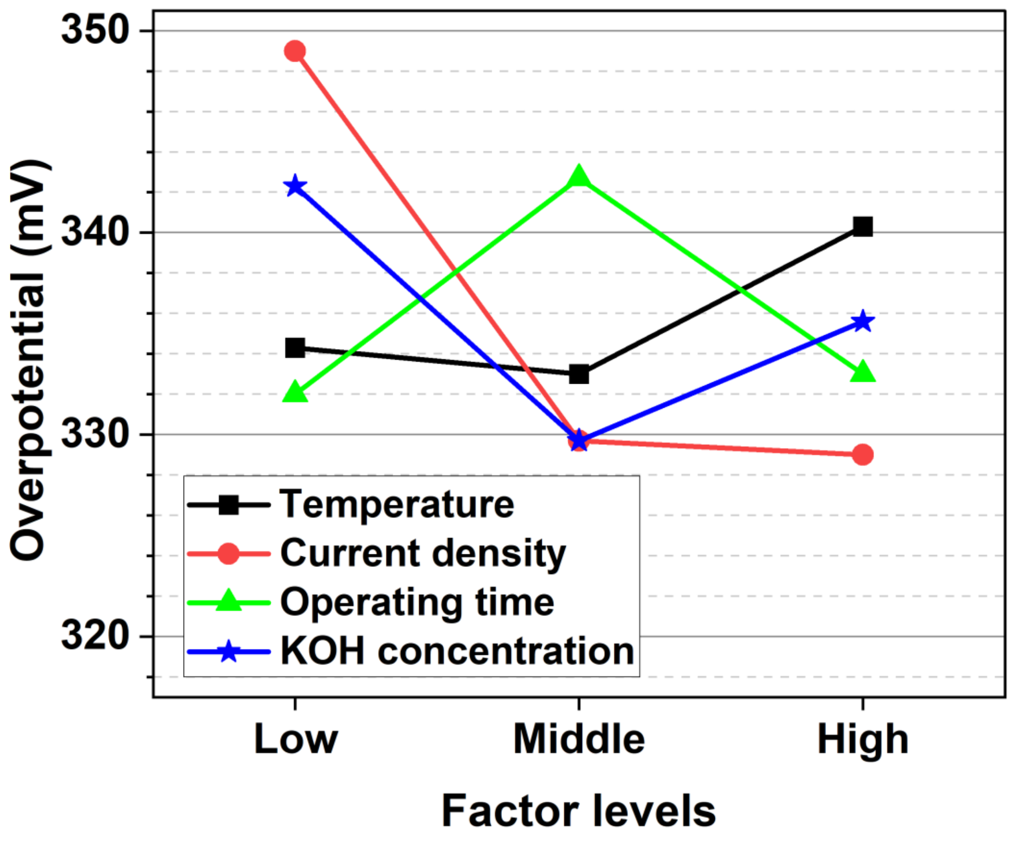


**Supplementary Figure S2**. The statistical analysis of factors on the OER activity effect based on the response of overpotential at 100 mA $cm^{-2}$. The lowest overpotential corresponds to the optimum conditions. The supposed optimal activation conditions are middle temperature and KOH concentration, low operating time, and high current density.

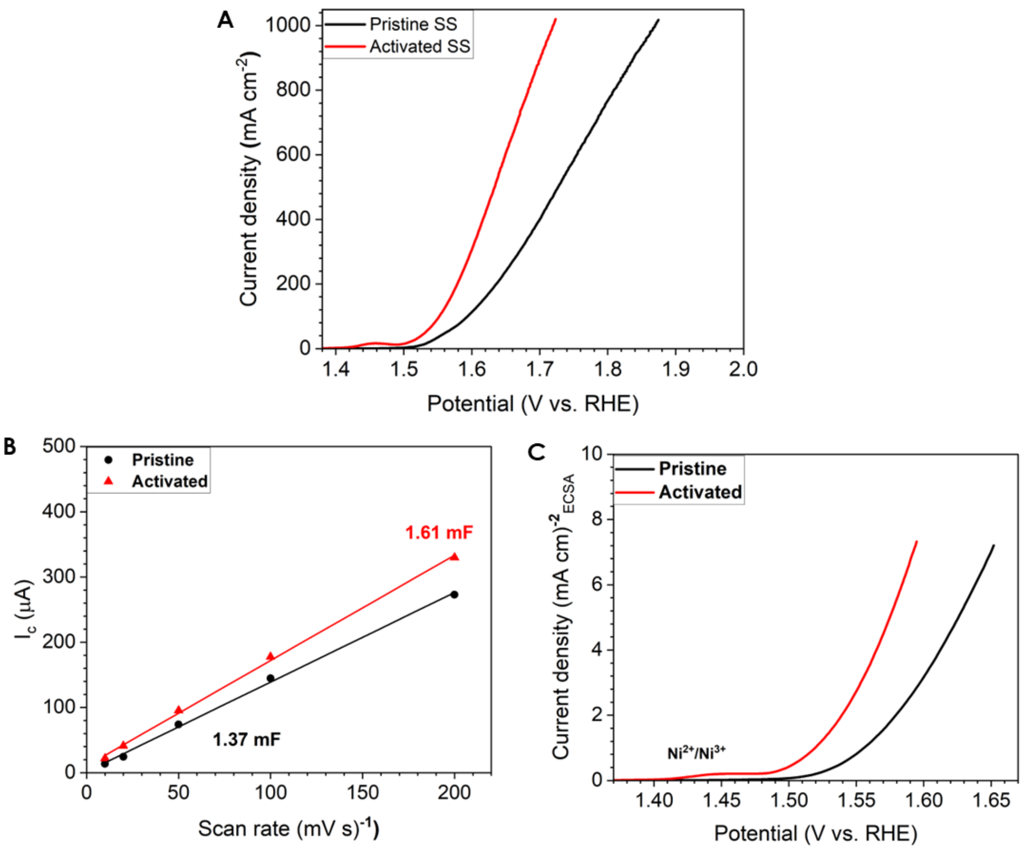


**Supplementary Figure S3.** The Linear sweep voltammetry (LSV) curves at high current density, and the electrochemically active surface (ECSA) measurement with the corresponding normalized LSV. (a) LSV curves of pristine and the activated SS electrodes at high current. (b) Double-layer capacitance ($C_{dl}$) measurement of pristine and activated SS electrodes. (c) The corresponding normalized LSV.

*For estimating the ECSA of the SS, a specific capacitance of 0.04 mF $cm^{-2}$ was employed according to the literature report[1]. The ECSAs of the activated and pristine SS electrodes are 34.3 and 40.3 $cm^{-2}$, respectively, indicating the ECSA increases after the activation.

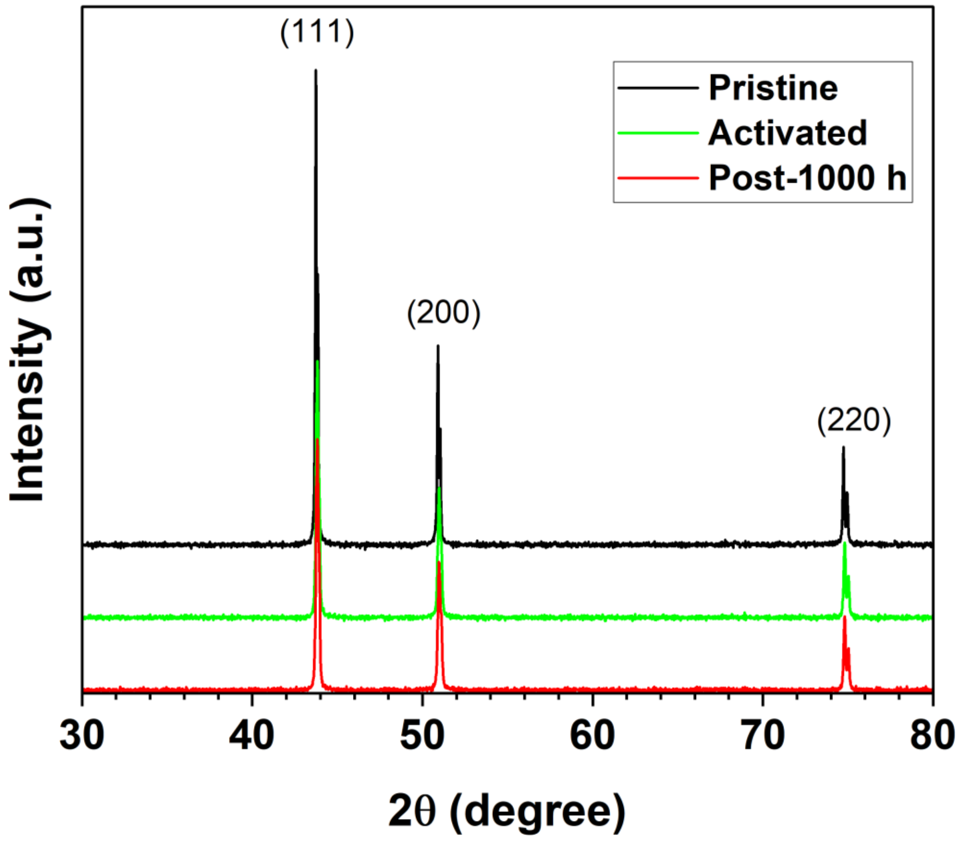


**Supplementary Figure S4**.XRD patterns of pristine, activated, and post-1000h SS electrodes. No obvious difference is noticed in XRD patterns among those three SS electrodes.

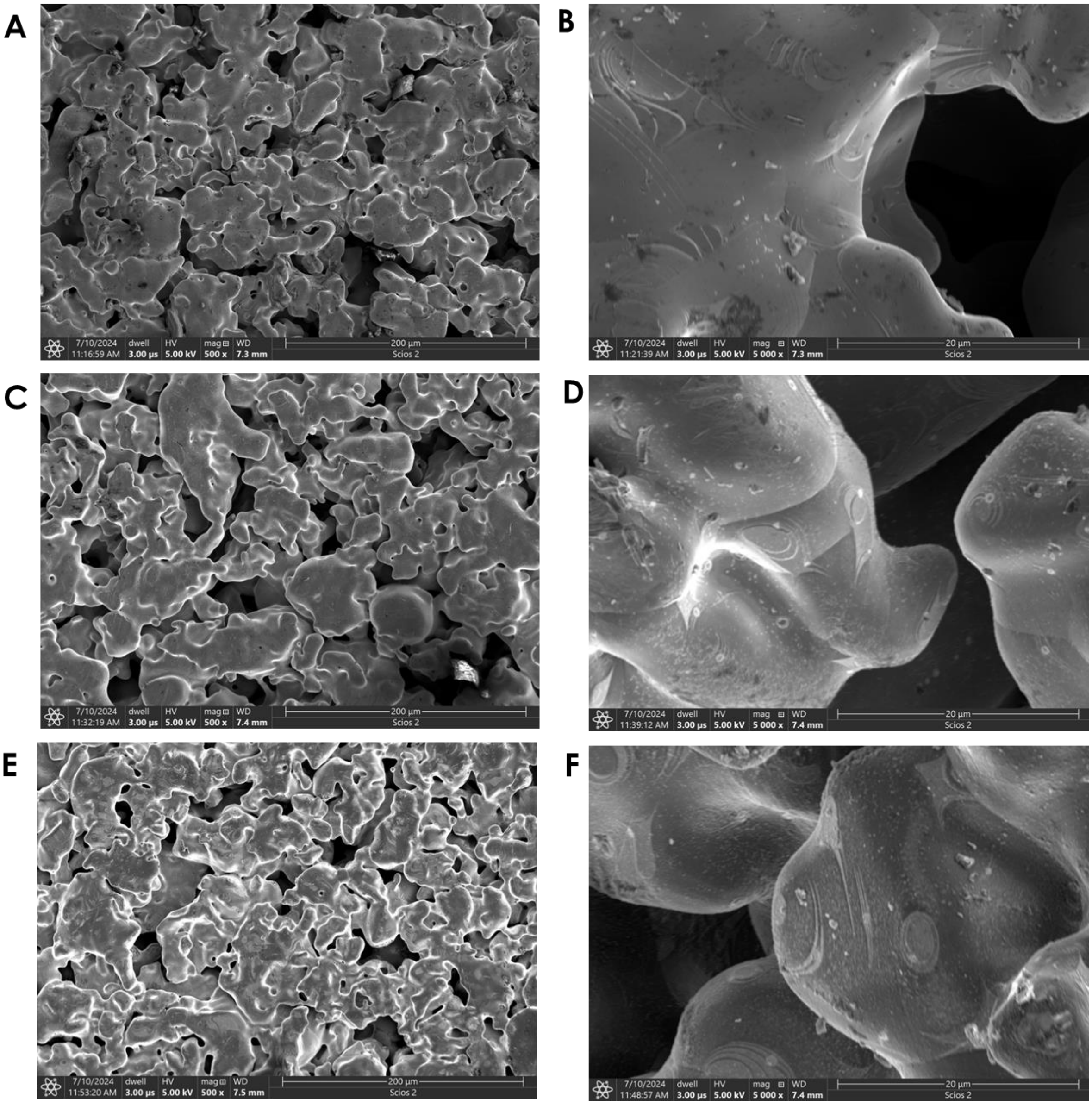


**Supplementary Figure S5**. **SEM images**. (a) and (b) pristine, (c) and (d) activated, and (e) and (f) post-1000 h SS electrodes. No big difference was observed among those three SS electrodes.

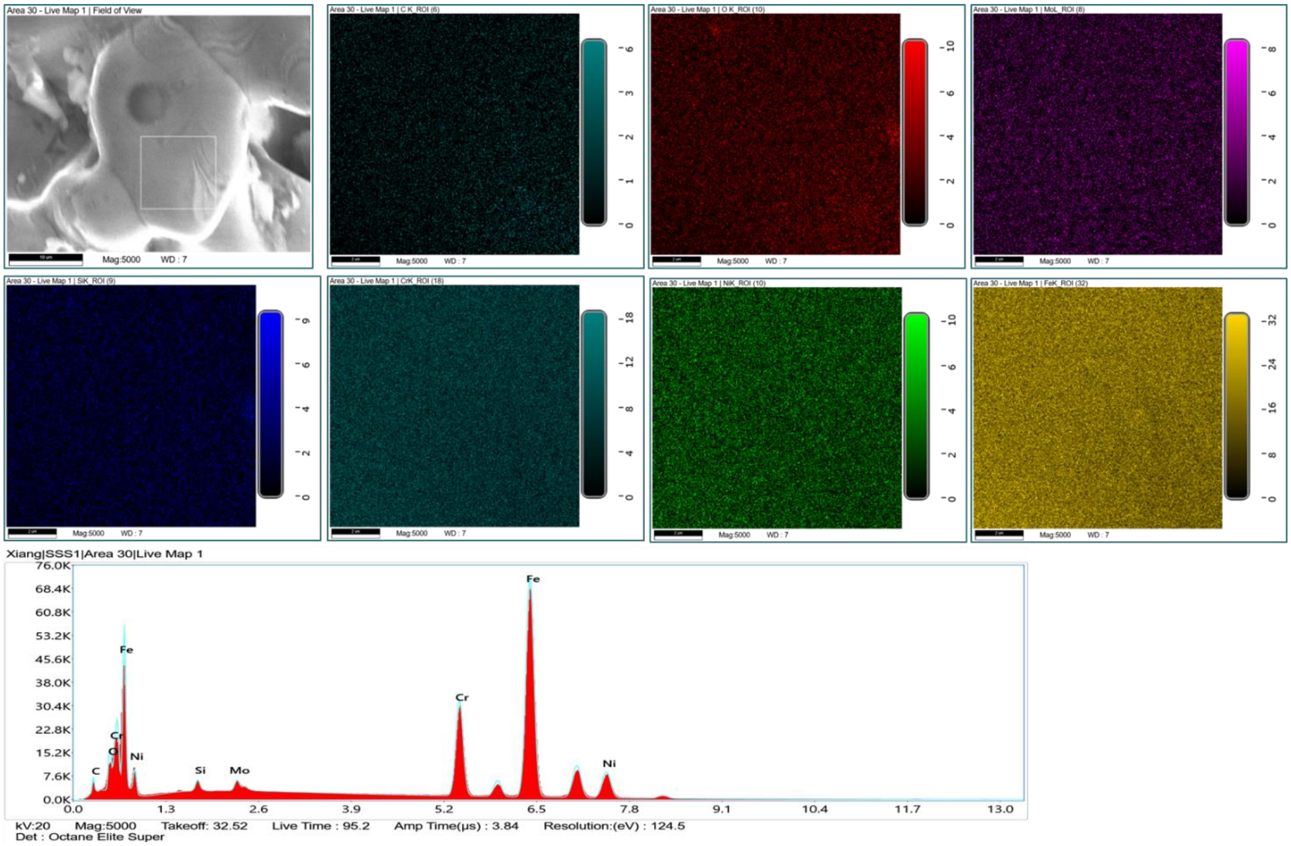


**Supplementary Figure S6**. EDS characterization of pristine SS electrode surface.

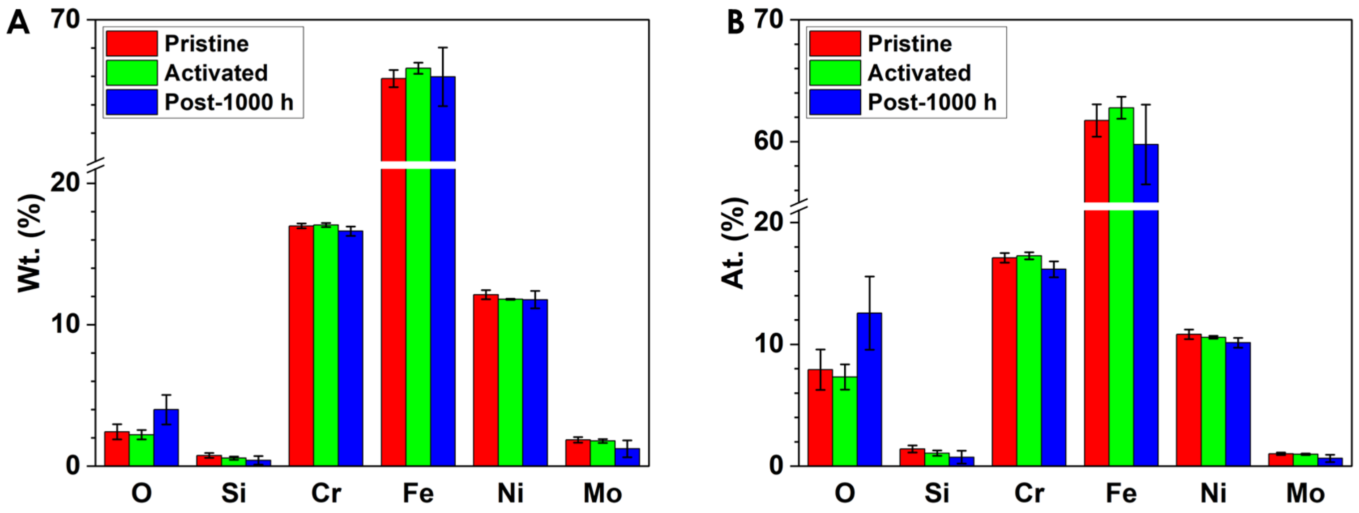


**Supplementary Figure S7**. EDS analysis for the Fe, Cr, Ni, and O on the electrode surface. (a) weight percentage and (b) atom percentage. No big difference in metal composition change is observed among those three electrodes if we consider the error bars, while the O content increased after the durability test.

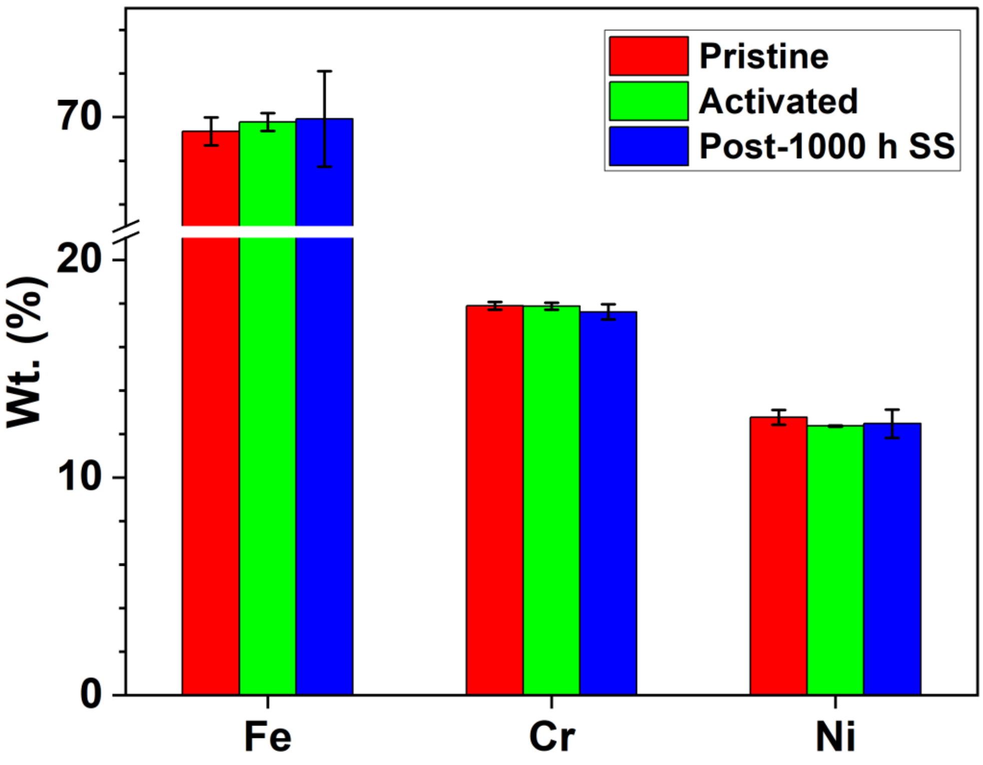


**Supplementary Figure S8**. EDS summary of Fe, Cr, and Ni for the pristine, activated, post-1000 h durability SS electrodes.

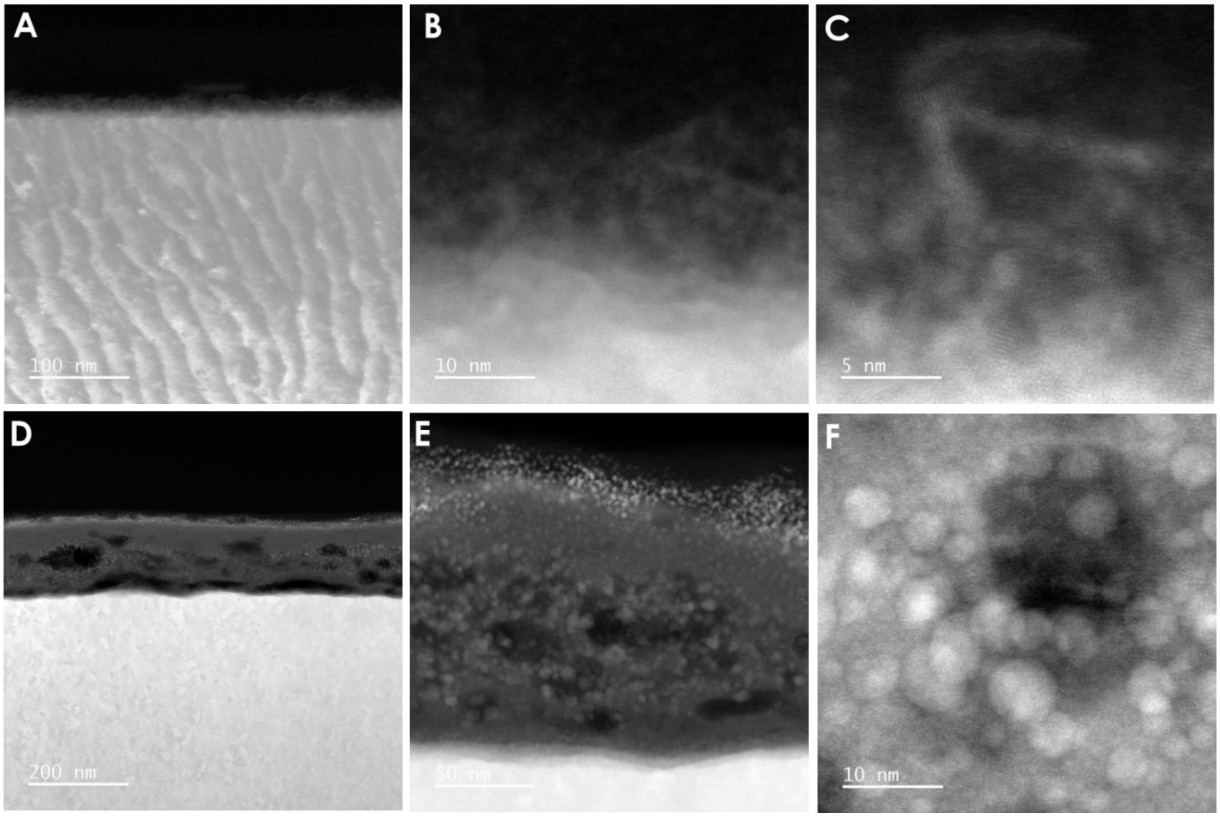


**Supplementary Figure S9**. HR-STEM cross-section images. (a)-(c) HR-STEM cross-section images of activated SS electrode with different magnifications. (d)-(f) HR-STEM cross-section images of post-1000 h SS electrode with different magnifications

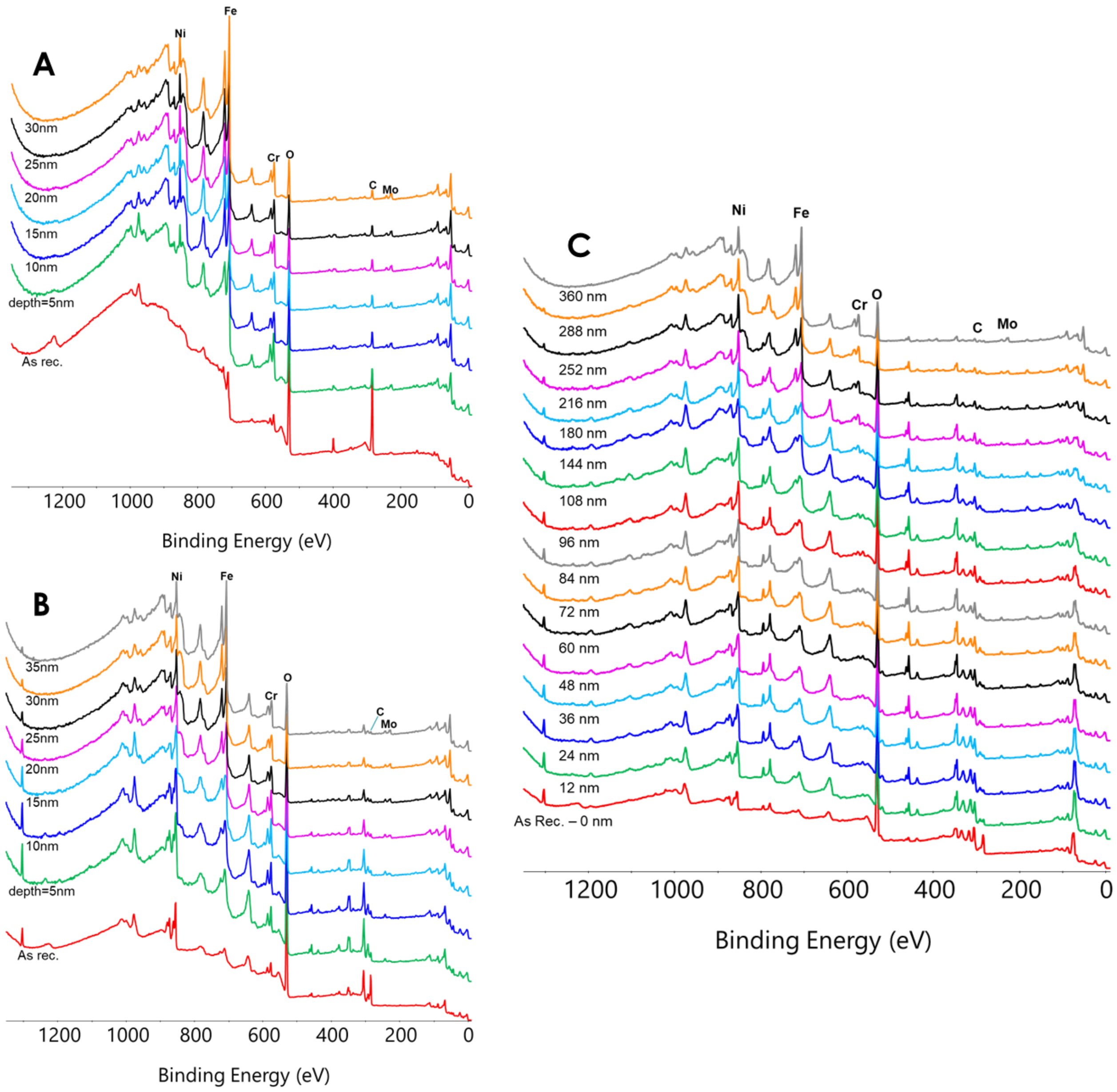


**Supplementary Figure S10**. XPS survey with different depths of pristine, activated, and post-1000 h SS electrodes. (a) Pristine SS electrode from surface to 30 nm. (b) Activated SS electrode from surface to 35 nm. (c) Post-1000 h SS electrode from surface to 360 nm.

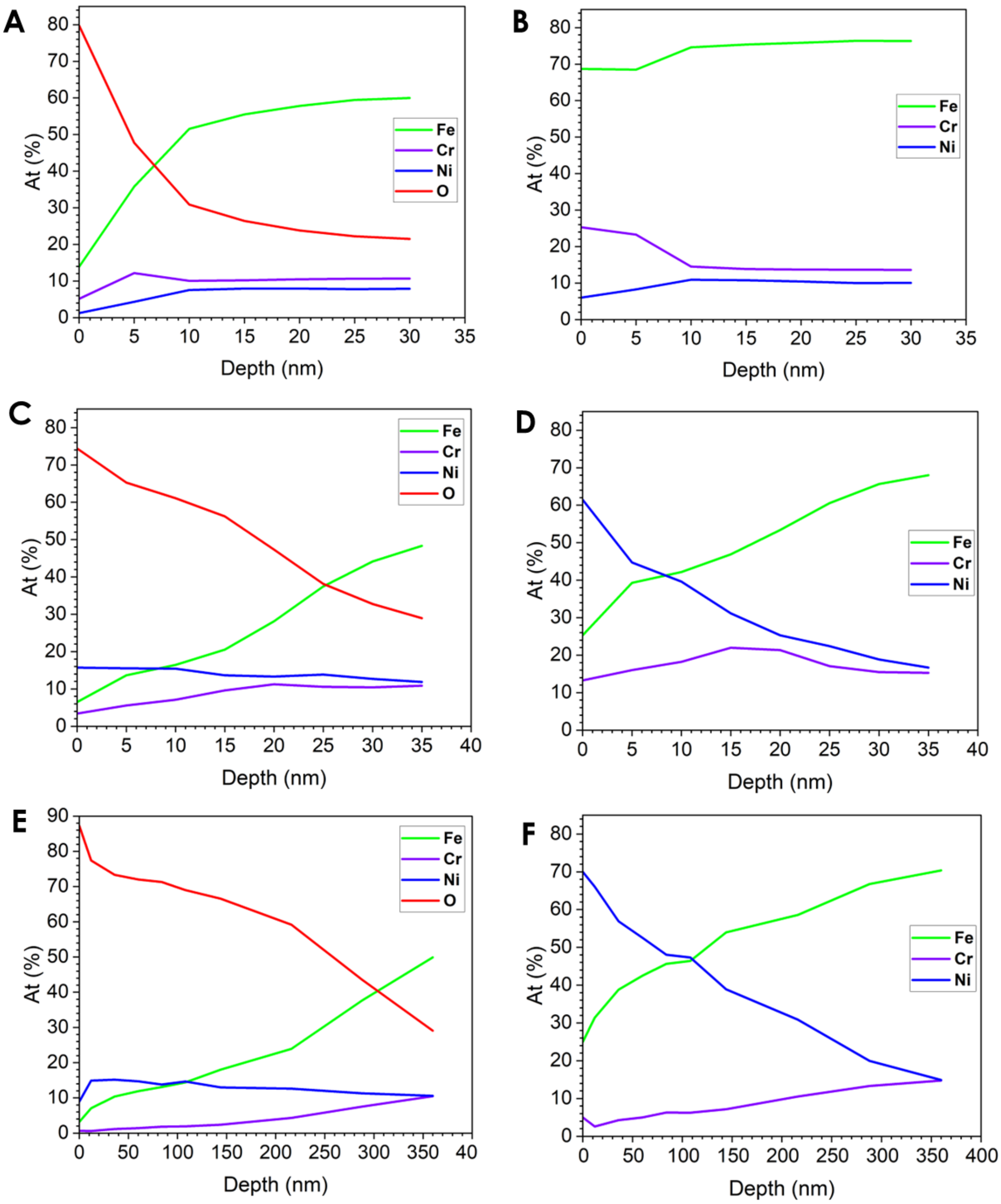


**Supplementary Figure S11**. Fe, Cr, Ni, O element compositions based on XPS surveys of pristine, activated, and post-1000 h SS electrodes corresponding to fig. S11. (a) and (b) Pristine SS electrode from surface to 30 nm. (c) and (d) Activated SS electrode from surface to 35 nm. (e) and (f) Post-1000 h SS electrode from surface to 360 nm.

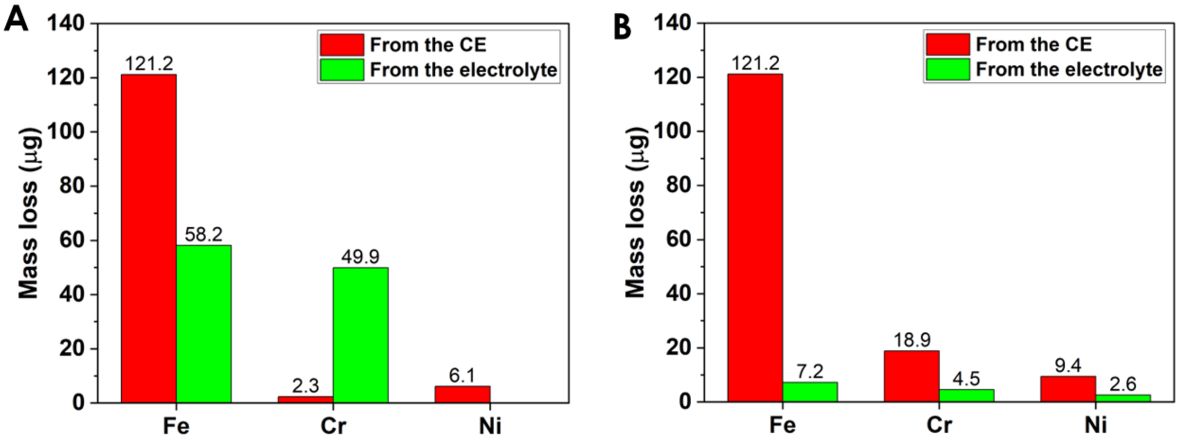


**Supplementary Figure S12**. The comparison of metal mass detected from the CE and electrolyte. (a) In the activation process of sample 6. (b) In the 1000 h durability test.

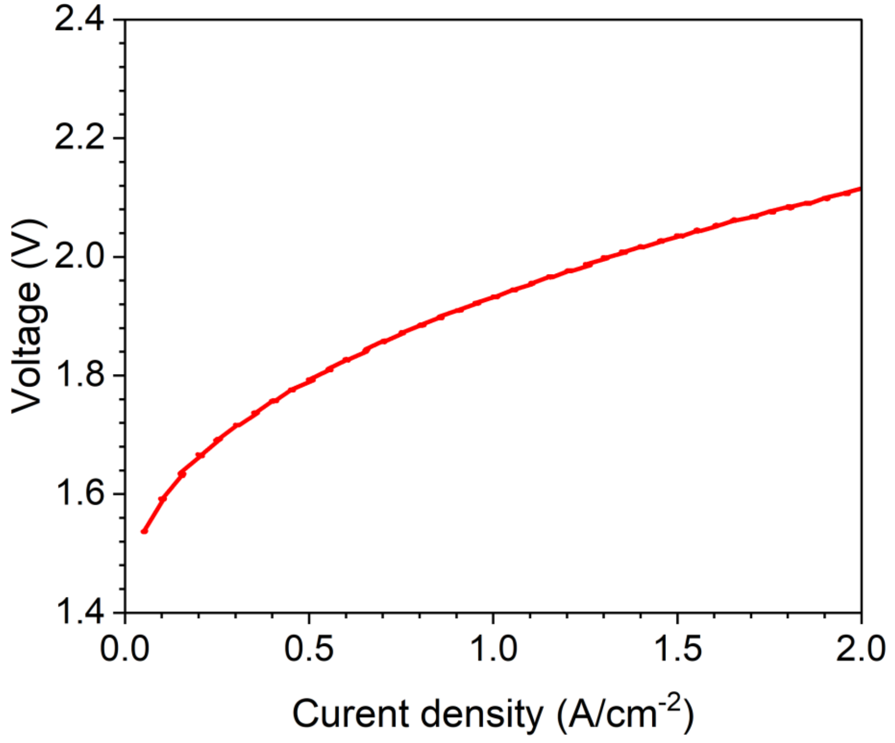


**Supplementary Figure S13**. Polarization curve of activated SS electrode in AEMWE.

**Supplementary Tables**

**Supplementary Table S1**. The orthogonal experimental design

| Trials* | Temperature (°C) | Current density ($mA\ cm^{-2}$) | Operating time (h) | KOH concentration (M) |
|---|---|---|---|---|
| 1 | 20 | 50 | 2 | 1 |
| 2 | 20 | 200 | 8 | 4 |
| 3 | 20 | 1000 | 5 | 7 |
| 4 | 50 | 50 | 8 | 7 |
| 5 | 50 | 200 | 5 | 1 |
| 6 | 50 | 1000 | 2 | 4 |
| 7 | 80 | 50 | 5 | 4 |
| 8 | 80 | 200 | 2 | 7 |
| 9 | 80 | 1000 | 8 | 1 |

*In the orthogonal experimental design, a total of 4 operating factors were investigated and optimized: the temperature, current density, operating time, and KOH concentration, and each factor has three levels: low, middle, and high. For the temperature, the low/middle/high are 20/50/80 °C; for the current density, the low/middle/high are 50/200/1000 mA $cm^{-2}$; for the operating time, the low/middle/high are 2/5/8 h; for the KOH concentration, the low/middle/high are 1/4/7 M.

**Supplementary Table S2**. The weight of 10 cut 316 SS electrodes

| **Samples** | 1 | 2 | 3 | 4 | 5 | 6 | 7 | 8 | 9 | 10 |
|---|---|---|---|---|---|---|---|---|---|---|
| **Weight* (mg)** | 265 | 267 | 268 | 265 | 267 | 268 | 265 | 267 | 265 | 264 |

*The average weight of 10 SS electrodes is 266.1 mg with a standard deviation of 1.4 mg (0.6% compared with the average weight), indicating great repeatability.

**Supplementary Table S3**. The overpotentials at current densities of 10 and 100 mA $cm^{-2}$

| Samples | Overpotential (mV, 10 mA $cm^{-2}$) | Overpotential (mV, 100 mA $cm^{-2}$) |
|---|---|---|
| Pristine* | 297 | 366 |
| 1 | 297 | 350 |
| 2 | 252 | 319 |
| 3 | 262 | 334 |
| 4 | 279 | 343 |
| 5 | 283 | 340 |
| 6 | 257 | 316 |
| 7 | 294 | 354 |
| 8 | 268 | 330 |
| 9 | 283 | 337 |

*SS electrode without activation. The activity was enhanced for all pretreated SS compared with the pristine, and sample 6 exhibits the highest activity with the overpotentials of 316 mV at the current densities of 100 mA $cm^{-2}$.

**Supplementary Table S4**. The comparison of some electrocatalysts toward OER based the on literature review

| Catalysts | Electrolyte | Overpotential @ 10 mA cm$^{-2}$ (mV) | Overpotential @ 100 mA cm$^{-2}$ (mV) | Tafel slope (mV dec$^{-1}$) | Stability (mA cm$^{-2}$$_{(h)}$) | Refs. |
|---|---|---|---|---|---|---|
| $Co_3C$ | 1 M NaOH | 455 | N/A | N/A | N/A | 2 |
| CoxNi-LDHs | 1 M KOH | 290 | N/A | 65 | $100_{(40)}$ | 3 |
| $La(CrMnFeCo_2Ni)O_3$ | 1 M NaOH | 325 | N/A | 51 | $10_{(50)}$ | 4 |
| $Fe_xNi_{1-x}OOH$ | 1 M KOH | 325 | N/A | 65 | N/A | 5 |
| Ni NP/NiFe | 1 M KOH | 328 | N/A | 62 | $10_{(20)}$ | 6 |
| NiCd/Fe | 0.1 M NaOH | 290 | N/A | 38 | N/A | 7 |
| $Fe_xNi_{9-X}S_8$ | 1 M KOH | 354 | $420_{(100)}$ | 56 | $10_{(10)}$ | 8 |
| $IrO_2$ | 1M KOH | 345 | $434_{(100)}$ | 58 | $10_{(10)}$ | 8 |
| FeNi-MOF | 0.1 M KOH | 270 | N/A | 47 | $20_{(15)}$ | 9 |
| CoOOH-NS | 1 M KOH | 253 | N/A | N/A | $10_{(100)}$ | 10 |
| $NiCo_{1.75}Fe_{0.25}O_4$@NiO @NF | 1 M KOH | N/A | $272_{(100)}$ | 54 | $50_{(12)}$ | 11 |
| VCoCOx@NF | 1 M KOH | 240 | N/A | 64 | $10_{(70)}$ | 12 |
| MoFe:NiOOH/$Ni(OH)_2$ | 1 M KOH | N/A | $280_{(100)}$ | 47 | $100_{(50)}$ | 13 |
| $FeO_xH_y$-Ni | 1 M KOH | 277 | N/A | N/A | N/A | 14 |
| CoFeNiCrMn | 1M KOH | 307 | N/A | 34.7 | $10_{(168)}$ | 15 |
| hBN/$NiFeO_xH_y$ | 1 M KOH | 230 | N/A | 30 | $1000_{(150)}$ | 16 |
| $Ca_{0.8}Pr_{0.2}Co_{0.8}Fe_{0.2}O_{3-\delta}$ | 0.1 M KOH | 391 | N/A | 83 | N/A | 17 |
| $Ni^{vac}Fe^{vac}$-LDH | 1 M KOH | 230 | $359_{(100)}$ | 52 | N/A | 18 |
| NiFe-SE/CFP | 1 M KOH | 281 | N/A | 40.9 | $10_{(20)}$ | 19 |
| NiFe LDH-Ni(III)Li | 1 M KOH | 248 | N/A | 35 | $10_{(24)}$ | 20 |
| NiFeB-P MNs | 1 M KOH | 252 | N/A | 35.2 | $10_{(23)}$ | 21 |
| NiFe-2D MOF | 1 M KOH | 260 | N/A | 56 | $10_{(24)}$ | 22 |
| NiFeOOH | 1 M KOH | 346 | $500_{(100)}$ | 56.8 | N/A | 23 |
| $(Ni_7Fe_3)$OOH-S | 1 M KOH | 238 | $298_{(100)}$ | 42.7 | $500_{(400)}$ | 24 |
| Fe-ZnMOFS/NF | 1 M KOH | 240 | N/A | 34 | $10_{(800)}$ | 25 |
| Fe-$La_{0.5}Sr_{0.5}Co_{0.8}Fe_{0.2}O_3$ | 1 M KOH | 320 | N/A | N/A | N/A | 26 |
| Ni-riched SS | 1 M KOH | 257 | $316_{(100)}$ | 52.1 | $1000_{(1000)}$ | This work |

**Supplementary Table S5**. The effect analysis of 4 factors on OER activity corresponding to **Supplementary Figure S2**

| Factors | R-value* |
|---|---|
| Temperature | 7.3 |
| Current density | 19.3 |
| Operating time | 10.7 |
| KOH concentration | 12.7 |

*R-value is obtained from the subtraction between the biggest and the smallest value from Fig. S2, and a larger R-value indicates a stronger effect. Thus, the current density has a significant impact on the activity, the KOH concentration and operating time show intermediate influence, and the temperature exhibits the weakest effect.

**Supplementary Table S6**. The Fe, Cr, and Ni determination from the counter electrode (CE) in the activation and durability processes

| Trials* | Fe (ppm) | Cr (ppm) | Ni (ppm) |
|---|---|---|---|
| Blank 1 M HCl | 0.48 | 0.002 | 0.007 |
| Sample 6 (Activation) | 12.6 | 0.232 | 0.615 |
| Durability | 12.6 | 1.89 | 0.949 |

*Fe, Cr, and Ni metals were determined from the counter electrode (CE) washed in 10 ml 1 M HCl, and all reported data here excluding the impurities from the blank 1 M HCl solution.

**Supplementary Table S7**. The Fe, Cr, and Ni determination from the electrolyte in the activation and durability processes

| Trials* | Fe (ppm) | Cr (ppm) | Ni (ppm) |
|---|---|---|---|
| Blank 1 M KOH | 0.0354 | 0.0039 | <0.0043 |
| Sample 6 (Activation) | 0.1300 | 0.105 | <0.0043 |
| 100 h | 0.0285 | 0.0033 | <0.0043 |
| 500 h | 0.0497 | 0.0075 | <0.0043 |
| 1000 h | 0.0956 | 0.0414 | 0.0214 |

*Fe, Cr, and Ni metals were determined in 120 ml KOH electrolyte, and all reported data here excluding the impurities from the blank KOH solution.

**Supplementary Table S8**. The total of Fe, Cr, and Ni loss in the in activation and durability processes

| Trials* | Fe (μg) | Cr (μg) | Ni (μg) | Total loss (μg) | Ratio (Fe/Ni) | Ratio (Cr/Ni) | Ratio ((Fe/Cr) |
|---|---|---|---|---|---|---|---|
| Sample 6 (Activation) | 179.4 | 52.2 | 6.1 | 237.7 | 29.5 | 8.6 | 3.4 |
| 1000 h durability | 128.4 | 23.4 | 12.0 | 163.8 | 10.7 | 2.0 | 5.5 |

*****According to the mass loss ratio results, the Fe and Cr dissolved much faster than Ni in the activation stage.

## Supplementary References


1 McCrory, C. C., Jung, S., Peters, J. C. & Jaramillo, T. F. Benchmarking heterogeneous electrocatalysts for the oxygen evolution reaction. *Journal of the American Chemical Society* **135**, 16977-16987, (2013).

2 Kim, J.-H. *et al.* Transformation of a Cobalt Carbide (Co3C) Oxygen Evolution Precatalyst. *ACS Applied Energy Materials* **1**, 5145-5150, (2018).

3 Hu, W., Liu, Q., Lv, T., Zhou, F. & Zhong, Y. Impact of interfacial CoOOH on OER catalytic activities and electrochemical behaviors of bimetallic CoxNi-LDH nanosheet catalysts. *Electrochimica Acta* **381**, 138276, (2021).

4 Nguyen, T. X., Liao, Y.-C., Lin, C.-C., Su, Y.-H. & Ting, J.-M. Advanced High Entropy Perovskite Oxide Electrocatalyst for Oxygen Evolution Reaction. *Advanced Functional Materials* **31**, 2101632, (2021).

5 Swierk, J. R., Klaus, S., Trotochaud, L., Bell, A. T. & Tilley, T. D. Electrochemical Study of the Energetics of the Oxygen Evolution Reaction at Nickel Iron (Oxy)Hydroxide Catalysts. *The Journal of Physical Chemistry C* **119**, 19022-19029, (2015).

6 Gao, X., Long, X., Yu, H., Pan, X. & Yi, Z. Ni Nanoparticles Decorated NiFe Layered Double Hydroxide as Bifunctional Electrochemical Catalyst. *Journal of The Electrochemical Society* **164**, H307, (2017).

7 Kim, J.-H. *et al.* An active nanoporous Ni(Fe) OER electrocatalyst via selective dissolution of Cd in alkaline media. *Applied Catalysis B: Environmental* **225**, 1-7, (2018).

8 Amin, H. M. A., Attia, M., Tetzlaff, D. & Apfel, U.-P. Tailoring the Electrocatalytic Activity of Pentlandite FexNi9-XS8 Nanoparticles via Variation of the Fe : Ni Ratio for Enhanced Water Oxidation. *ChemElectroChem* **8**, 3863-3874, (2021).

9 Wang, L. *et al.* Fe/Ni Metal–Organic Frameworks and Their Binder-Free Thin Films for Efficient Oxygen Evolution with Low Overpotential. *ACS Applied Materials & Interfaces* **8**, 16736-16743, (2016).

10 Zhou, J. *et al.* Electrochemically accessing ultrathin Co (oxy)-hydroxide nanosheets and operando identifying their active phase for the oxygen evolution reaction. *Energy & Environmental Science* **12**, 739-746, (2019).

11 Upale, P., Verma, S. & Ogale, S. B. Superior oxygen evolution reaction performance of NiCoFe spinel oxide nanowires in situ grown on β-Ni(OH)2 nanosheet-decorated Ni foam: case studies on stoichiometric and off-stoichiometric oxides. *Journal of Materials Chemistry A* **11**, 8972-8987, (2023).

12 Meena, A. *et al.* Bifunctional oxovanadate doped cobalt carbonate for high-efficient overall water splitting in alkaline-anion-exchange-membrane water-electrolyzer. *Chemical Engineering Journal* **430**, 132623, (2022).

13 Jin, Y., Huang, S., Yue, X., Du, H. & Shen, P. K. Mo- and Fe-Modified Ni(OH)2/NiOOH Nanosheets as Highly Active and Stable Electrocatalysts for Oxygen Evolution Reaction. *ACS Catalysis* **8**, 2359-2363, (2018).

14 Jiang, Q. *et al.* Active oxygen species mediate the iron-promoting electrocatalysis of oxygen evolution reaction on metal oxyhydroxides. *Nature Communications* **14**, 6826, (2023).

15 Baek, J. *et al.* Synergistic effects of mixing and strain in high entropy spinel oxides for oxygen evolution reaction. *Nature Communications* **14**, 5936, (2023).

16 Lu, Y. *et al.* One-atom-thick hexagonal boron nitride co-catalyst for enhanced oxygen evolution reactions. *Nature Communications* **14**, 6965, (2023).

17 Moon, J. *et al.* Active learning guides discovery of a champion four-metal perovskite oxide for oxygen evolution electrocatalysis. *Nature Materials*, (2023).

18 Peng, L. *et al.* Atomic Cation-Vacancy Engineering of NiFe-Layered Double Hydroxides for Improved Activity and Stability towards the Oxygen Evolution Reaction. *Angewandte Chemie International Edition* **60**, 24612-24619, (2021).

19 Guo, Y. *et al.* Metal–Organic Framework-Derived Bimetallic NiFe Selenide Electrocatalysts with Multiple Phases for Efficient Oxygen Evolution Reaction. *ACS Sustainable Chemistry & Engineering* **9**, 2047-2056, (2021).

20 Xu, Z. *et al.* Engineering NiFe layered double hydroxide by valence control and intermediate stabilization toward the oxygen evolution reaction. *Journal of Materials Chemistry A* **8**, 26130-26138, (2020).

21 Kang, Y. *et al.* Soft Template-Based Synthesis of Mesoporous Phosphorus- and Boron-Codoped NiFe-Based Alloys for Efficient Oxygen Evolution Reaction. *Small* **18**, 2203411, (2022).

22 Liu, Y. *et al.* Freestanding 2D NiFe Metal–Organic Framework Nanosheets: Facilitating Proton Transfer via Organic Ligands for Efficient Oxygen Evolution Reaction. *Small* **18**, 2201076, (2022).

23 Hashemi, N., Nandy, S., Chae, K. H. & Najafpour, M. M. Anodization of a NiFe Foam: An Efficient and Stable Electrode for Oxygen-Evolution Reaction. *ACS Applied Energy Materials* **5**, 11098-11112, (2022).

24 Liu, W. *et al.* Electrochemical hydroxidation of sulfide for preparing sulfur-doped NiFe (oxy) hydroxide towards efficient oxygen evolution reaction. *Chemical Engineering Journal* **454**, 140030, (2023).

25 Wang, Y., Zhao, L., Ma, J. & Zhang, J. Confined interface transformation of metal–organic frameworks for highly efficient oxygen evolution reactions. *Energy & Environmental Science* **15**, 3830-3841, (2022).

26 Zhao, J.-W. *et al.* Key roles of surface Fe sites and Sr vacancies in the perovskite for an efficient oxygen evolution reaction via lattice oxygen oxidation. *Energy & Environmental Science* **15**, 3912-3922, (2022).
